# Supplementary material for: A host–guest semibiological photosynthesis system coupling artificial and natural enzymes for solar alcohol splitting
Source: Nat Commun. 2021 Aug 24;12:5092. doi: 10.1038/s41467-021-25362-4 (PMC8384870; doi:10.1038/s41467-021-25362-4)
Supplement: Supplementary file 1 — Supplementary information [file 41467_2021_25362_MOESM1_ESM.pdf]

Supplementary information

A host-guest semibiological photosynthesis system coupling artificial and natural enzymes for solar alcohol splitting

Junkai Cai[1], Liang Zhao*[1], Cheng He[1], Yanan Li[1], and Chunying Duan*[1]

[1]State Key Laboratory of Fine Chemicals, Zhang Dayu School of Chemistry, Dalian University of Technology, Dalian 116024, People's Republic of China.

*Corresponding Authors. E-mail: zhaol@dlut.edu.cn; cyduan@dlut.edu.cn.

Supplementary Methods

Experimental materials

All chemicals were of reagent grade quality obtained from commercial sources, biomaterial ADH from Saccharomyces cerevisiae was purchased from Sigma-Aldrich. Unless stated otherwise, all operations were carried out under an atmosphere of dry argon using Schlenk and vacuum techniques. Solvents were dried by standard methods and freshly distilled prior to use. NMR spectra were measured on a Bruker 400M spectrometer with chemical shifts reported as ppm (in DMSO-d_6 or $CDCl_3$, TMS as internal standard). ESI-MS spectra were carried out on HPLC-Q-Tof MS spectrometer. The fluorescent spectra were measured on Edinburgh FS-1000. UV-Vis spectra were measured on a HP 8453 spectrometer. CD spectra were measured on a JASCO J-810 spectropolarimeter. Dynamic light scattering (DLS) measurements were performed on Malvern Zetasizer Nano ZS90 analyzer. EPR spectra were measured on a Bruker E500 spectrometer. ITC essay were measured on a TA Nano−ITC Instruments. The elemental analyses of C, H and N were performed on a Vario EL III elemental analyzer. All electrochemical measurements were carried out under Ar at room temperature and performed on a ZAHNER ENNIUM electrochemical workstation with a conventional three-electrode system with an Ag/AgCl electrode as a reference electrode, a platinum silk with 0.5 mM diameter as a counter electrode, and glassy carbon electrode as a working electrode. Gel filtration chromatography were performed on AKTA purifier 100 using a sephadex G-75 gel sieving column, UV-Vis detector (detection wavelength 280 nm), 1×PBS as mobile phase.

Experimental method

General method for photocatalytic proton reduction. Varying amounts of the catalyst and **PNQ** was added into an EtOH/H_2O solution (v:v = 3:2, pH 4.5, 5.0 mL) containing NADH with a magnetic stir bar. The flask was sealed with a septum and protected from air by Ar. The samples were irradiated by a 300 W Xenon lamp. The reaction was maintained at 25ºC by using a water filter to absorb heat.

General method for photocatalytic alcohol splitting. Varying amounts of the

catalyst, ADH and **PNQ** was added into an EtOH/H_2O solution (v:v = 3:2, pH 4.5, 5.0 mL) containing NAD^+ with a magnetic stir bar. The flask was sealed with a septum and protected from air by Ar. The samples were irradiated by a 300 W Xenon lamp. The reaction was maintained at 25°C by using a water filter to absorb heat.

The generated hydrogen was characterized by GC 7890T instrument analysis using a 5 Å molecular sieve column, thermal conductivity detector, and argon used as carrier gas. The amount of hydrogen generated was determined by the external standard method[1]. The generated aldehyde was characterized by an Agilent 6890N GC using a FFAP capillary column, flame ionization detector, and nitrogen used as carrier gas. The amount of aldehyde generated was determined by the external standard method[2,3].

For normal homogenous system, the quenching behavior was evaluated by the Stern-Volmer equation as in Supplementary Equation (1)[4]:

$$\frac{F_0}{F} = 1 + k_q\tau_0[\mathrm{Q}] = 1 + K_{\mathrm{SV}}[\mathrm{Q}] \tag{1}$$

F_0 and F are the emission intensity in the absence and presence of quencher, respectively, k_q is the quenching rate constant, τ_0 is the excited-state lifetime in the absence of quencher, and $[Q]$ is the concentration of quencher. Plotting the ratio F_0/F against the quencher concentration thus gives a straight line having a y-intercept equal to 1 and a slope, termed the Stern-Volmer constant (K_{SV}), equal to $k_q\tau_0$.

For supramolecular system, the binding behavior showed in UV-Vis spectra was evaluated by the 1:1 binding model, as in Supplementary Equation (2)[5]:

$$A - A_0 = \frac{1}{2}(\varepsilon_{\mathrm{HG}} - \varepsilon_{\mathrm{H}})\left[\left([G_0] + [H_0] + \frac{1}{K_{\mathrm{a}}}\right) - \sqrt{\left([G_0] + [H_0] + \frac{1}{K_{\mathrm{a}}}\right)^2 - 4[H_0][G_0]}\right] \tag{2}$$

A_0 and A are the absorbance in the absence and presence of guest, respectively, $\varepsilon_{\mathrm{HG}}$ is the molar absorptivity of the host-guest species, ε_{H} is the molar absorptivity of the free host H, $[G_0]$ is the total concentration of the guest, $[H_0]$ is the the initial concentration of the host, K_a is the association constant.

The host-guest binding behavior showed in fluorescence spectra was evaluated by non-linear Hill plot, as in Supplementary Equation (3)[6]:

$$F - F_0 = \frac{1}{2}(F_L - F_0)\left[\left(\frac{[G_0]}{[H_0]} + 1 + \frac{1}{K_{\mathrm{a}}[H_0]}\right) - \sqrt{\left(\frac{[G_0]}{[H_0]} + 1 + \frac{1}{K_{\mathrm{a}}[H_0]}\right)^2 - \frac{4[G_0]}{[H_0]}}\right] \tag{3}$$

F_0 and F are the emission intensity in the absence and presence of guest,

respectively, F_L is the emission intensity of saturated value in presence of excess guest. $[G_0]$ is the total concentration of the guest, $[H_0]$ is the initial concentration of the host, K_a is the association constant.

Following is the formula derivation:

$$\mathrm{H} + \mathrm{G} \rightleftharpoons \mathrm{H{\cdot}G}$$

Generally, for the formation of 1:1 host-guest species H·G formed by host (H) and guest (G), if we assume that $[H_0]$ is the initial concentration of the host, the concentration $x[H_0]$ of H·G produces when adding the molar concentration $[G_0]$ of guest. K_a can be calculated by Supplementary Equation (4):

$$K_a = \frac{x}{(1-x)([G_0]-x[H_0])} \tag{4}$$

The quadratic equation can be rearranged to Supplementary Equation (5):

$$x^2 - \left(\frac{[G_0]}{[H_0]} + 1 + \frac{1}{K_a[H_0]}\right)x + \frac{[G_0]}{[H_0]} = 0 \tag{5}$$

The corresponding solution was shown in Supplementary Equation (6):

$$x = \frac{1}{2}\left[\left(\frac{[G_0]}{[H_0]} + 1 + \frac{1}{K_a[H_0]}\right) - \sqrt{\left(\frac{[G_0]}{[H_0]} + 1 + \frac{1}{K_a[H_0]}\right)^2 - \frac{4[G_0]}{[H_0]}}\right] \tag{6}$$

The measurements are performed under the conditions where the emission intensity of the free host in such a concentration is F_0; after addition of a given amount $[G_0]$, the fluorescent intensity can be defined by Supplementary Equation (7):

$$F = F_0(1-x) + F_L x \tag{7}$$

F_L is the emission intensity of saturated value in presence of excess guest. The equation can be rearranged to Supplementary Equation (8):

$$x = \frac{F - F_0}{F_L - F_0} \tag{8}$$

Combining Supplementary Equation (6) and (8) can afford Supplementary Equation (3).

Preparation and Characterizations

The photosensitizer 2-phenyl-4-(1-naphthyl)-quinolinium (**PNQ**) and catalyst Co**BDT**$_2$ were synthesized according to the reported procedures by S. Fukuzumi[7] and R. Eisenberg[8], respectively. And ligand H_6**TPS** was synthesized similar to the reported procedures and shown below[9,10].

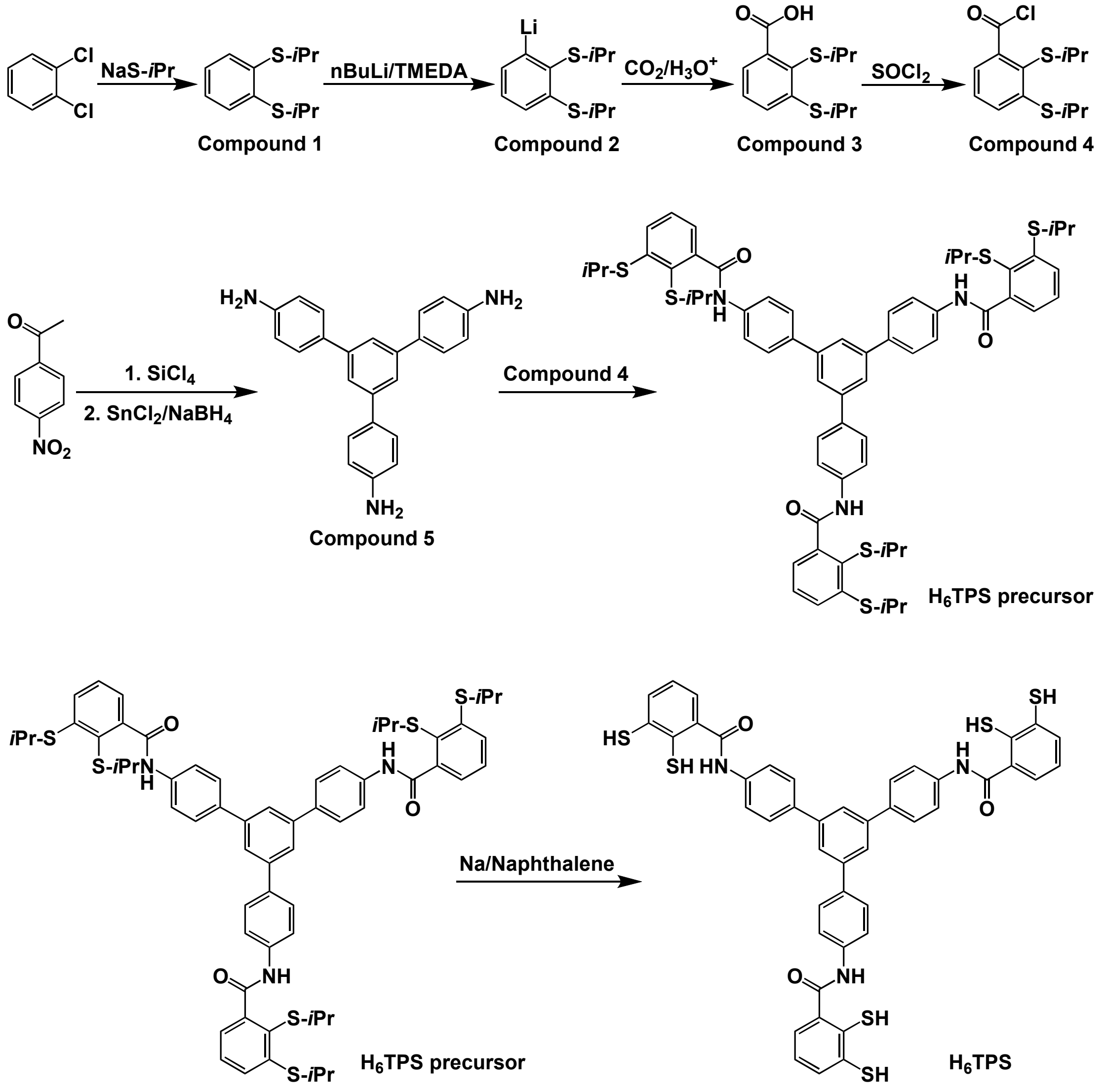

Supplementary Figure 1. The synthetic route of ligand H_6**TPS**.

Synthesis of compound **1**

1,2-Dichlorobenzene (10.7 g, 71.0 mmol) was added dropwise to a suspension of sodium isopropylmercaptane (35.0 g, 356.0 mmol) in DMF (200 mL). The mixture was stirred for 3 days at 100°C and was then allowed to cool to ambient temperature.

It was then poured into water (500 mL) and extracted with diethyl ether (3 × 100 mL). The combined organic layers were dried over Na_2SO_4 and filtered. Evaporation of the filtrate and evaporate under vacuum yielded compound **1** as a yellow oil, the crude product was purified by column chromatography (SiO_2, petrol ether), and the colorless oil compound **1** was obtained. Yield: 13.7 g, 85% (based on 1,2-dichloro benzene). ^{1}H NMR ($CDCl_3$, 400 MHz, ppm): δ 7.33 (m, 2H; ArH), 7.14 (m, 2H; ArH), 3.48 (m, 2H; SCH), 1.38 (d, J = 6.6 Hz, 6H; CH_3), 1.27 (d, J = 6.6 Hz, 6H; CH_3). ^{13}C NMR ($CDCl_3$, 101 MHz, ppm): δ 137.5, 130.6, 126.3, 36.8, 22.8.

Synthesis of compound **3**

A sample of n-butyllithium (8.8 mL of a 2.5 M solution in hexane, 22.0 mmol) was added dropwise to a solution of TMEDA (3.3 mL, 22.0 mmol) and compound **1** (5.0 g, 22.0 mmol) in hexane (200 mL) at 0ºC. After 1 h, the ice bath was removed and the stirring was continued at ambient temperature overnight to result in the formation of yellow slurry of compound **2**, which was used directly without further purification. Dry CO_2 was bubbled through a slurry of compound **2** (22.0 mmol) in hexane at 0ºC for 2 h. After evaporation of the solvent, the residue was dissolved in water and acidified with hydrochloric acid (37%) to pH 2. The aqueous solution was extracted with diethyl ether (3 × 100 mL) and the combined organic layers were dried over Na_2SO_4. Volatiles were removed in vacuum to afford a yellow solid. The product was recrystallized from hexane to yield an off-white powder. Yield: 3.4 g, 57% (based on **1**). ^{1}H NMR ($CDCl_3$, 400 MHz, ppm): δ 12.24 (s, 1H; CO_2H), 7.66 (dd, J = 3.6 Hz, J = 2.4 Hz, 1H; ArH), 7.38 (m, 2H; ArH), 3.54 (m, 2H; SCH), 1.38 (d, J = 6.6 Hz, 6H; CH_3), 1.27 (d, J = 6.6 Hz, 6H; CH_3). ^{13}C NMR ($CDCl_3$, 101 MHz, ppm): δ 171.3, 146.1, 137.8, 129.9, 129.4, 128.8, 126.1, 40.4, 36.1, 22.8, 22.4.

Synthesis of compound **5**

$SiCl_4$ (20.0 mL, 0.18 mol) was added dropwise to a solution of *p*-nitroaceto-phenone (10.0 g, 0.06 mol) in ethanol (60 mL) at 0ºC and then the mixture was refluxed for 10 h. After the mixture was cooled to room temperature, saturated NH_4Cl

(100 mL) was added and stirred for 30 min. The obtained yellow precipitate was filtered and dried, and added DMF (120 mL) directly with $SnCl_2·2H_2O$ (45.1 g, 0.20 mol). Then, $NaBH_4$ (2.3 g, 60.0 mmol) in 10 mL DMF was added dropwise to the solution and heated at 60ºC for 10 h under Ar. The reaction solution was cooled to room temperature and poured into ice, which adjusted to pH 9 and the precipitate was collected. The solid was recrystallized in ethanol and dried at vacuum and finally obtained white solid. Yield: 5.6 g, 80%. ^{1}H NMR ($CDCl_3$, 400 MHz): δ 7.59 (s, 3H; ArH), 7.50 (m, 6H; ArH), 6.78 (m, 6H; ArH), 3.73 (s, 6H; NH_2). ^{13}C NMR ($CDCl_3$, 101 MHz, ppm): δ 146.8, 142.3, 131.7, 128.4, 122.8, 115.5.

Synthesis of H_6**TPS** precursor

Thionyl chloride (1.0 mL) were added to a solution of compound **3** (1.1 g, 4.0 mmol) in chloroform (10 mL). The reaction mixture was heated under reflux conditions for 3 h, and then the solvent was removed in vacuum for compound **4**. The freshly prepared compound **4** was dissolved in THF (20 mL) directly and this solution was added to a solution of compound **5** (0.5 g, 1.30 mmol) and NEt_3 (2.0 mmol) in THF (40 mL) at 0ºC. Then, the reaction mixture was stirred for 12 h at ambient temperature. Subsequently, insoluble material was removed by filtration and the solvent was removed from the filtrate under vacuum. The pure product was obtained after washing with diethyl ether. Yield: 1.4 g, 92%. ^{1}H NMR ($CDCl_3$, 400 MHz, ppm): δ 9.07 (s, 3H; NH), 7.80-7.64 (m, 18H; ArH), 7.42-7.35 (m, 6H; ArH), 3.55-3.41 (m, 6H; $(CH_3)_2$), 1.41 (d, J = 6.7 Hz, 18H; CH_3), 1.24 (d, J = 6.7 Hz, 18H; CH_3). ^{13}C NMR ($CDCl_3$, 101 MHz, ppm): δ 166.1, 146.0, 142.0, 141.8, 137.6, 137.2, 129.2, 128.9, 128.5, 128.0, 126.4, 124.5, 120.3, 41.4, 36.3, 23.1, 22.7. ESI-MS calcd for $C_{63}H_{69}N_3O_3S_6$: 1107.37, found 1108.37 $[M+H]^+$, 1130.36 $[M+Na]^+$. Elemental analysis calcd for $C_{63}H_{69}N_3O_3S_6$: H, 6.27; C, 68.25; N, 3.79%; found: H, 6.34; C, 67.66; N, 3.75%.

Preparation of Co_3**TPS**$_2$

Dry, freshly distilled THF (20 mL) was added to a mixture of precursor (387.6

mg, 0.35 mmol), sodium (181.1 mg, 7.85 mmol), and naphthalene (336.5 mg, 2.60 mmol). The reaction mixture was stirred for 12 h at 25ºC. Subsequently methanol (5.0 mL) was added to remove unreacted sodium. The solvents were then removed under vacuum. The solid residue was dissolved in degassed water and the resulting solution was washed three times with degassed diethyl ether (3 × 20 mL). The aqueous solution was filtered and HCl (37%) was added dropwise until a white precipitate formed. This precipitate was isolated by filtration and washed with water and diethyl ether. The solid residue was dried under vacuum to give ligand H_6**TPS** as white powder, which was used directly without further purification for subsequent reaction.

A DMF solution (10 mL) of $Co(BF_4)_2 \cdot 6H_2O$ (183.9 mg, 0.54 mmol) was added with stirring to a DMF solution (20 mL) of H_6**TPS** and NaOH (85.6 mg, 2.14 mmol). A deep blue solution developed immediately and it was left to stir overnight. To this solution, NEt_4Cl (89.5 mg, 0.54 mmol) was added, and the solution was allowed to stir at room temperature for 4 h. Then, the solution poured into 200 mL diethyl ether, a dark blue precipitate formed. The solid was collected and re-dissolved in DMF, dark blue crystals of Co_3**TPS**$_2$ suitable for single crystal X-ray diffraction were obtained by diffusing diethyl ether into the DMF solution, yield: 51%. Elemental analysis calcd for $Co_3(C_{45}H_{27}N_3O_3S_6)_2 \cdot (NC_8H_{20})_3 \cdot (C_3H_7NO)$: H, 5.21; C, 60.03; N, 5.98%; found: H, 5.31; C, 59.96; N, 6.21%. ESI-MS: *m*/*z* = 625.6254 [Co_3(**TPS**)$_2$]$^{3-}$, 949.9336 [$NaCo_3$(**TPS**)$_2$]$^{2-}$.

Single Crystal X-ray Crystallography

Intensities of the Co_3**TPS**$_2$ were collected at 180(2) K on a Bruker SMART APEX CCD diffractometer equipped with graphite monochromated Mo-Kα (λ = 0.71073 Å) using the SMART and SAINT programs[11,12]. The structure was solved by direct methods and refined on F^2 by full-matrix least-squares methods with SHELXTL *version* 5.1 software[13].

Crystal data of Co_3**TPS**$_2$: $Co_3(C_{45}H_{27}N_3O_3S_6)_2 \cdot 3NC_8H_{20} \cdot C_3H_7NO \cdot 4.5H_2O$, M = 2421.81, Triclinic, space group $P-1$, black blue rod, a = 12.8740(12), b = 27.316(3), c = 27.453(4) Å, α = 119.649(3), β = 98.301(6), γ = 94.063(4), V = 8187.8(15) Å^3, Z = 2, Dc = 0.982 g cm^{-3}, μ(Mo-Kα) = 0.499 mm^{-1}, T = 180(2) K. 28559 unique reflections [R_{int} = 0.1044]. Final R_1 [with $I > 2\sigma(I)$] = 0.1074, wR_2 (all data) = 0.2388 for the data collected. CCDC number 2042990.

In the structural refinement of Co_3**TPS**$_2$, all the non-hydrogen atoms were refined anisotropically. Hydrogen atoms within the ligand backbones, a DMF molecule and three Et_4N^+ cations were fixed geometrically at calculated distances and allowed to ride on the parent non-hydrogen atoms. To assist the stability of refinements, two amide groups on the ligands, a DMF molecule and two Et_4N^+ cations were limited to the desired position with rational thermal parameters by several restrains. One methyl on an Et_4N^+ cation, and a carbon atom and nitrogen atom on the DMF were disordered into two parts with *s.o.f* of each part being refined using free variables. The thermal parameters on adjacent atoms in all Et_4N^+ cations, a DMF molecule and some parts of ligands were restrained to be similar. In addition, the SQUEEZE subroutine in PLATON was used for refinements[14].

Despite rapid handling times and a low-temperature collection, the quality of data was less than ideal. We have tried our best to gain a better data, but failed. The reported herein are the best possible result obtained after an optimal exposure time. The crystal is poorly diffracting due to the low equality of the crystal, which leads to an Alert level A in checkcif report.

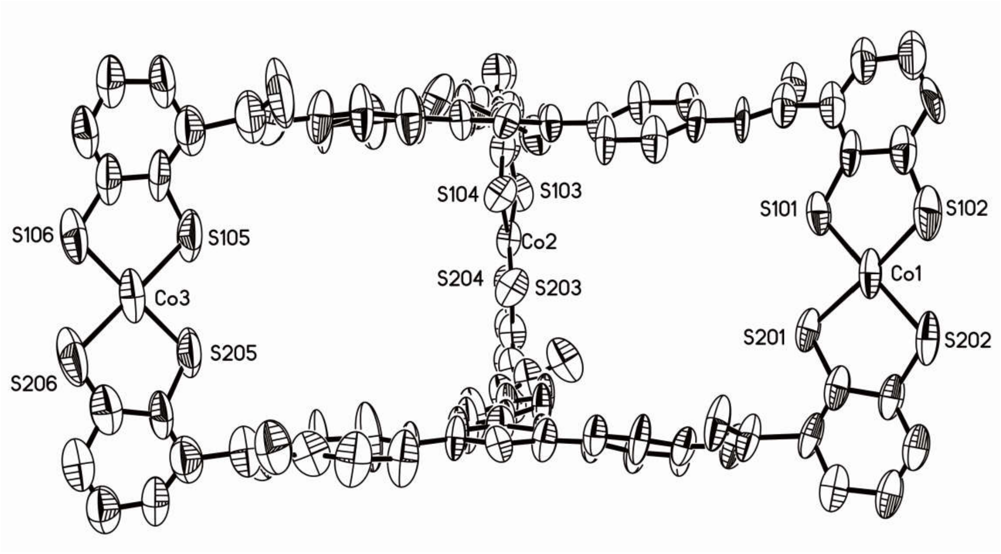

Supplementary Figure 2. A plot of the molecule triangular prism Co_3**TPS**$_2$ is shown, showing 30% probability displacement ellipsoids of non-hydrogen atoms. All hydrogen atoms are omitted for clarity.

Supplementary Table 1. Selective bond distance (Å) in Co_3**TPS**$_2$.

	bond distance (Å)		bond distance (Å)
Co(1)-S(101)	2.135(3)	S(101)-C(111)	1.739(13)
Co(1)-S(201)	2.155(3)	S(102)-C(112)	1.779(14)
Co(1)-S(102)	2.158(4)	S(103)-C(121)	1.691(13)
Co(1)-S(202)	2.171(3)	S(104)-C(122)	1.814(12)
Co(2)-S(203)	2.126(3)	S(105)-C(131)	1.744(15)
Co(2)-S(104)	2.127(3)	S(106)-C(132)	1.653(16)
Co(2)-S(103)	2.142(3)	S(201)-C(211)	1.650(12)
Co(2)-S(204)	2.155(3)	S(202)-C(212)	1.761(12)
Co(3)-S(205)	2.118(4)	S(203)-C(221)	1.755(13)
Co(3)-S(105)	2.143(4)	S(204)-C(222)	1.670(14)
Co(3)-S(206)	2.132(4)	S(205)-C(231)	1.735(14)
Co(3)-S(106)	2.148(4)	S(206)-C(232)	1.624(16)

Supplementary Table 2. Selective bond angle (°) in Co_3**TPS**$_2$.

	bond angle (°)		bond angle (°)
S(101)-Co(1)-S(201)	88.36(13)	C(122)-S(104)-Co(2)	105.2(5)
S(101)-Co(1)-S(102)	90.76(15)	C(131)-S(105)-Co(3)	106.8(5)
S(201)-Co(1)-S(102)	171.41(13)	C(132)-S(106)-Co(3)	103.8(6)
S(101)-Co(1)-S(202)	171.24(12)	C(211)-S(201)-Co(1)	103.9(5)
S(201)-Co(1)-S(202)	90.76(14)	C(212)-S(202)-Co(1)	105.4(4)
S(102)-Co(1)-S(202)	91.39(16)	C(221)-S(203)-Co(2)	104.8(5)
S(203)-Co(2)-S(104)	170.35(14)	C(222)-S(204)-Co(2)	102.2(5)
S(203)-Co(2)-S(103)	89.64(15)	C(231)-S(205)-Co(3)	109.4(5)
S(104)-Co(2)-S(103)	90.53(15)	C(232)-S(206)-Co(3)	102.0(6)
S(203)-Co(2)-S(204)	90.36(15)	C(116)-C(111)-S(101)	126.2(11)
S(104)-Co(2)-S(204)	90.74(16)	C(112)-C(111)-S(101)	119.1(11)
S(103)-Co(2)-S(204)	172.42(13)	C(113)-C(112)-S(102)	124.3(11)
S(205)-Co(3)-S(105)	89.18(17)	C(111)-C(112)-S(102)	114.1(12)
S(205)-Co(3)-S(206)	91.03(19)	C(122)-C(121)-S(103)	123.5(14)
S(105)-Co(3)-S(206)	176.79(19)	C(126)-C(121)-S(103)	120.8(10)
S(205)-Co(3)-S(106)	178.18(17)	C(121)-C(122)-S(104)	115.3(13)
S(105)-Co(3)-S(106)	90.48(17)	C(123)-C(122)-S(104)	117.2(10)
S(206)-Co(3)-S(106)	89.41(18)	C(132)-C(131)-S(105)	112.9(14)
C(111)-S(101)-Co(1)	107.6(4)	C(136)-C(131)-S(105)	116.2(12)
C(112)-S(102)-Co(1)	108.1(5)	C(133)-C(132)-S(106)	122.2(14)
C(121)-S(103)-Co(2)	105.3(5)	C(131)-C(132)-S(106)	125.7(15)

ESI-MS Spectra

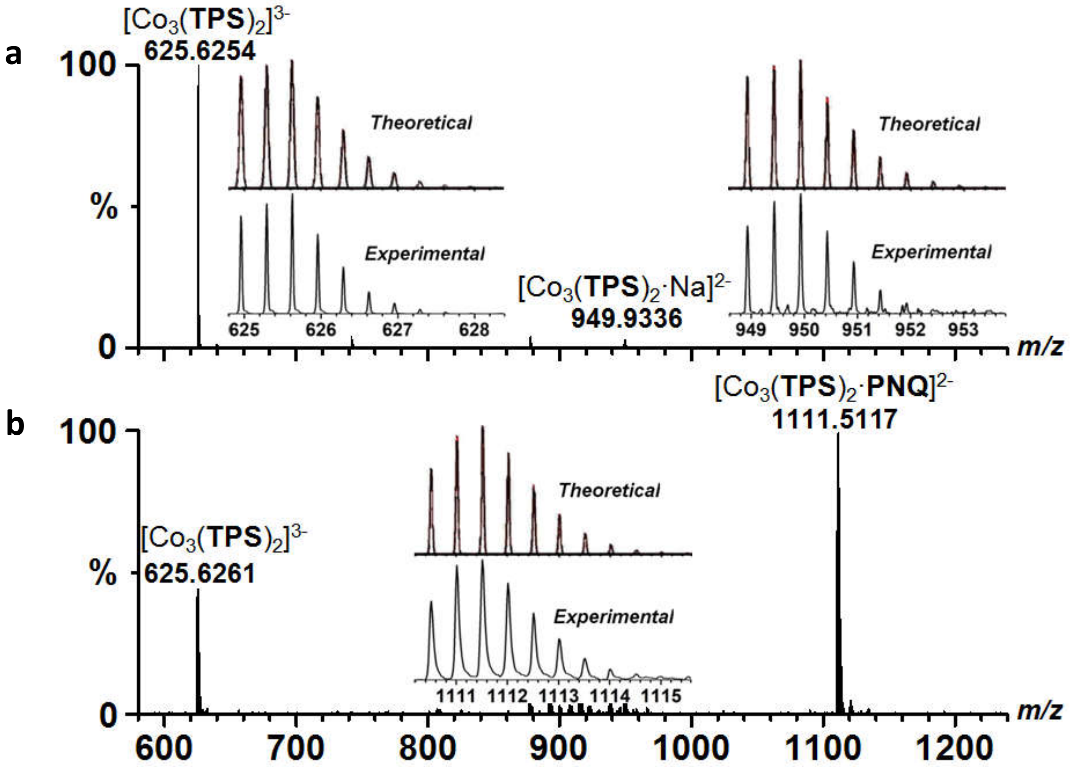

Peak	Value of m/z	Specie assigned
1	625.6254	$[Co_3(\mathbf{TPS})_2]^{3-}$
2	949.9336	$[Co_3(\mathbf{TPS})_2{\cdot}Na]^{2-}$
3	1111.5117	$[Co_3(\mathbf{TPS})_2{\cdot}\mathbf{PNQ}]^{2-}$

Supplementary Figure 3. ESI-MS spectra of 1.0 mM Co_3**TPS**$_2$ (a) and Co_3**TPS**$_2$ following the addition of 1.0 equiv of **PNQ** (b) in DMF solution. The inserts show the measured and simulated isotopic patterns at *m*/*z* = 625.6254, 949.9336 and 1111.5117, respectively.

Data for Spectral Titrations

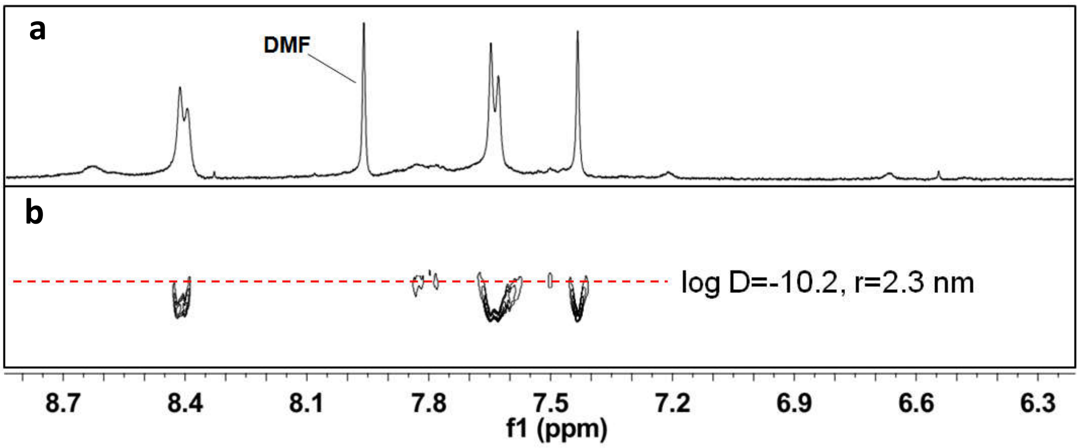

Supplementary Figure 4. (a) ^{1}H NMR spectra (aromatic region) of Co_3**TPS**$_2$ (1.0 mM) in d_6-DMSO. (b) Superposition of ^{1}H DOSY spectra of Co_3**TPS**$_2$ (1.0 mM) in d_6-DMSO.

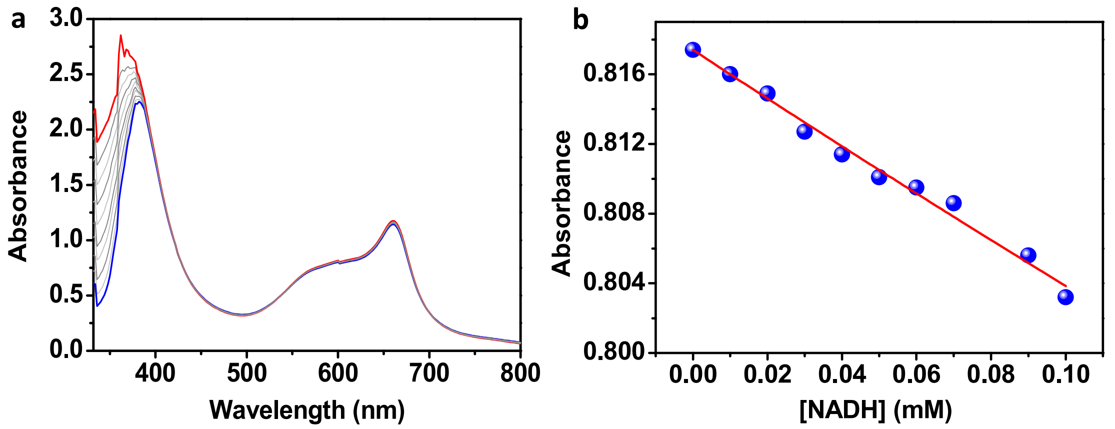

Supplementary Figure 5. (a) UV-Vis absorption spectra of **PNQ** (0.1 mM) and Co_3**TPS**$_2$ (0.1 mM) upon the addition of NADH (total 0.1 mM) in EtOH/H_2O (v:v = 3:2). (b) Plot of the absorbance at 660 nm with a nonlinear curve fit (1:1 binding model).

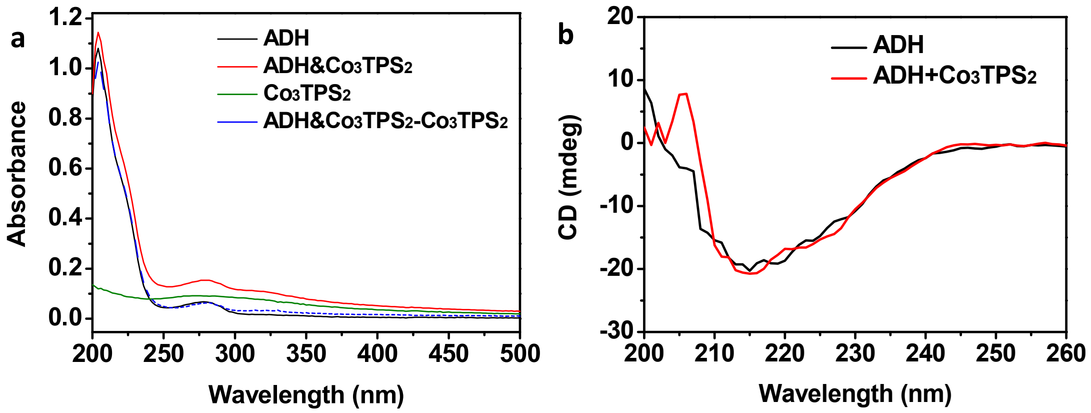

Supplementary Figure 6. (a) UV-Vis absorption spectra of ADH (1.0 μM), Co_3**TPS**$_2$ (1.0 μM) and ADH (1.0 μM) following the addition of 1.0 equiv of Co_3**TPS**$_2$ in EtOH/H_2O (v:v = 3:2). (b) CD spectra of ADH (1.0 μM) and ADH (1.0 μM) following the addition of 1.0 equiv of Co_3**TPS**$_2$ in EtOH/H_2O (v:v = 3:2).

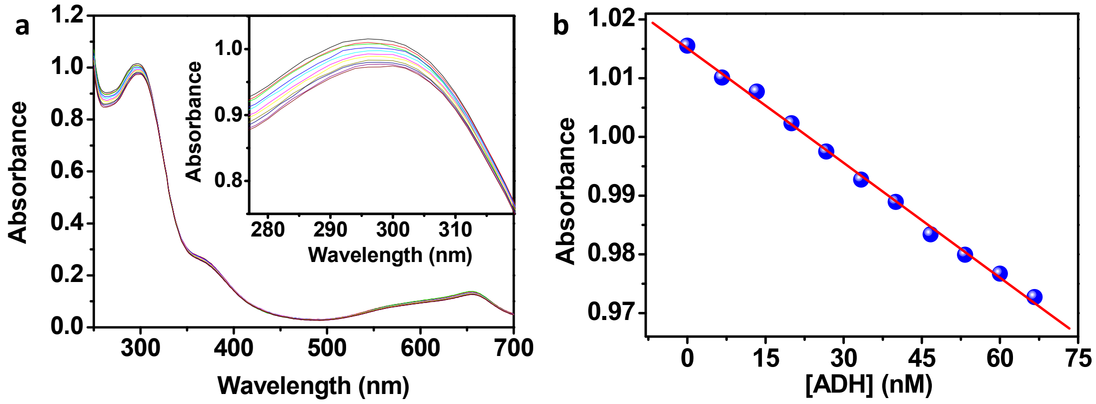

Supplementary Figure 7. (a) UV-Vis absorption spectra of Co_3**TPS**$_2$ (10.0 μM) in EtOH/H_2O (v:v = 3:2) upon addition of ADH. (b) The dependence of the absorbance at 300 nm on ADH concentration.

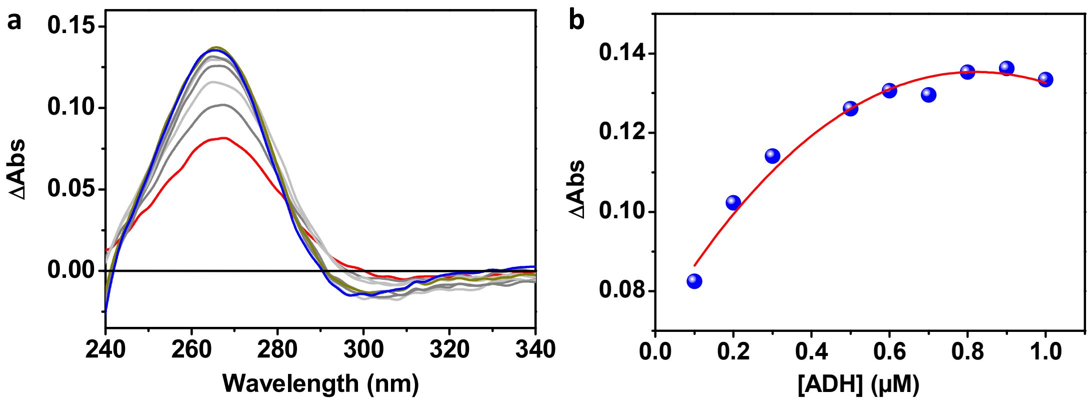

Supplementary Figure 8. (a) UV-Vis absorption difference spectra of Co_3**TPS**$_2$ (1.0 μM) in EtOH/H_2O (v:v = 3:2) upon addition of ADH (total 1.0 μM). (b) Plot of the absorbance changes at 268 nm with a nonlinear curve fit (1:1 binding model).

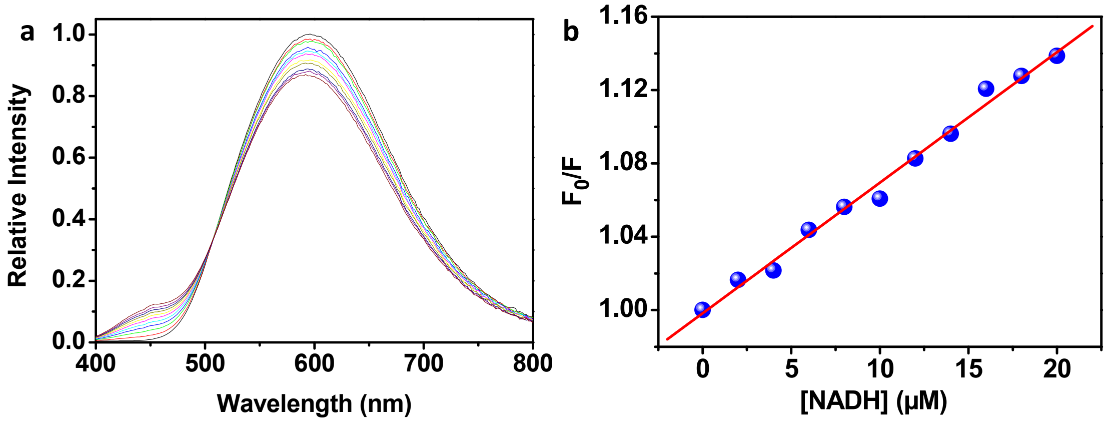

Supplementary Figure 9. (a) Luminescence spectra family of **PNQ** (10.0 μM) in EtOH/H_2O (v:v = 3:2) upon the addition of NADH. (b) The Stern-Volmer fitting of the titration curve at 594 nm.

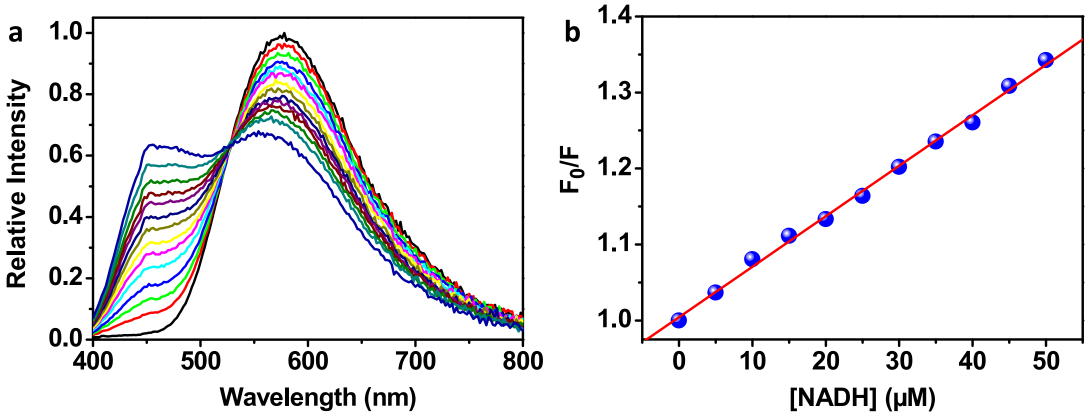

Supplementary Figure 10. (a) Luminescence spectra family of **PNQ** (0.1 mM) and Co**BDT**$_2$ (0.3 mM) in EtOH/H_2O (v:v = 3:2) upon the addition of NADH. (b) The Stern-Volmer fitting of the titration curve at 578 nm.

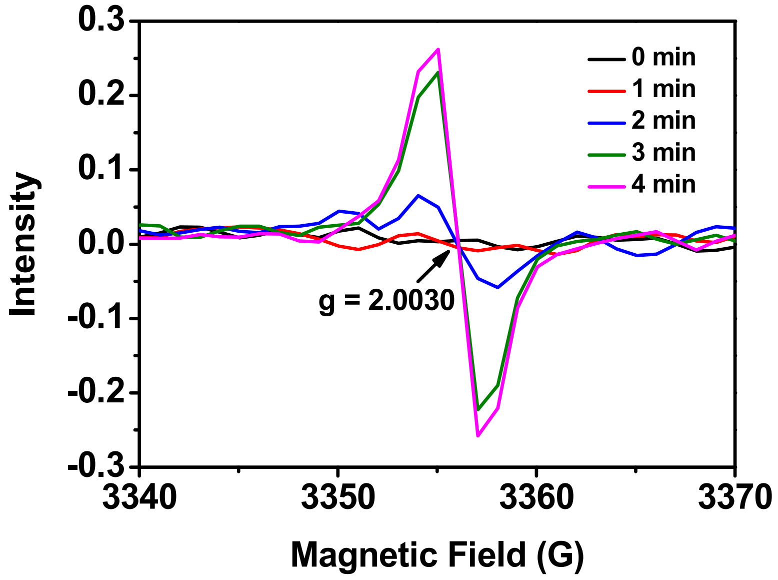

Supplementary Figure 11. EPR spectra collected at 233 K for the reaction system containing Co_3**TPS**$_2$ (20.0 μM), **PNQ** (0.5 mM) and NADH (2.0 mM) in DMF/H_2O (v:v = 3:2) upon the irradiation of Xenon lamp.

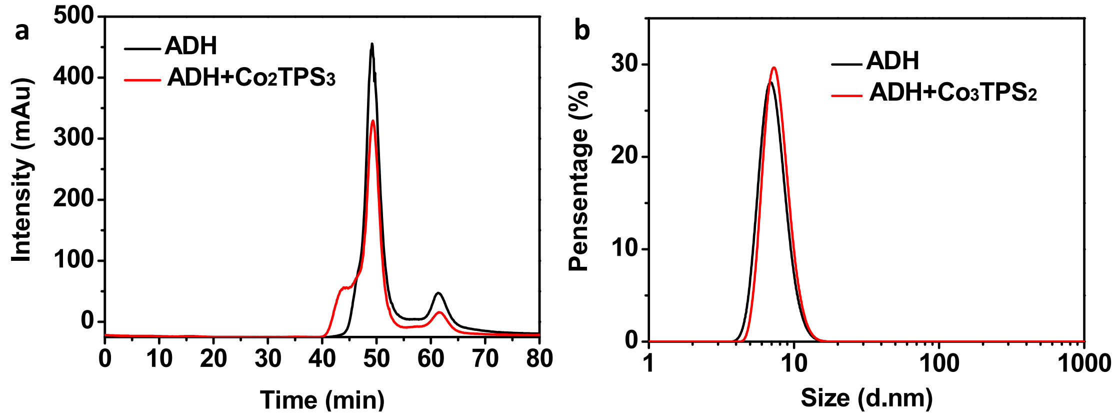

Supplementary Figure 12. (a) Gel filtration chromatography of 0.1 mM ADH and the mixture of 0.1 mM ADH and Co_3**TPS**$_2$ with 1×PBS as mobile phase. (b) DLS analysis of 0.1 mM ADH and the mixture of 0.1 mM ADH and Co_3**TPS**$_2$ in H_2O.

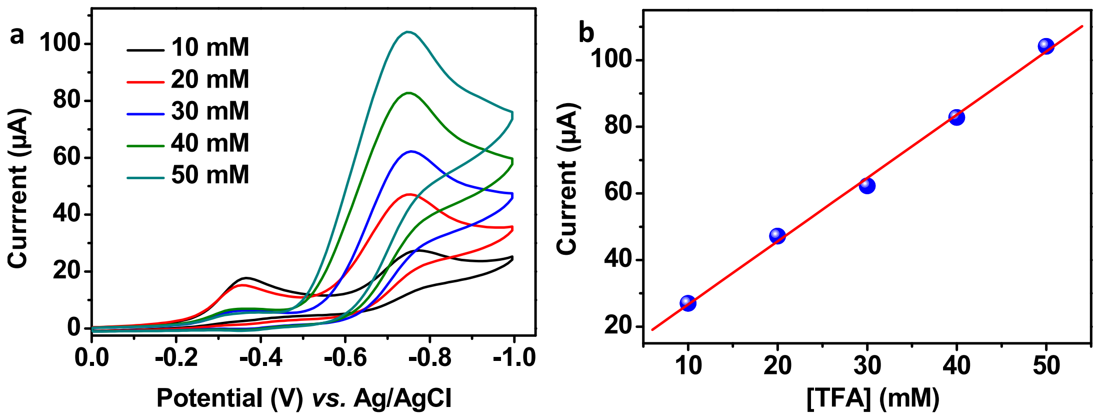

Supplementary Figure 13. (a) Cyclic voltammograms of 0.10 mM Co_3**TPS**$_2$ in DMF solution containing 0.1 M $TBAPF_6$ upon the addition of trifluoroacetic acid (TFA, 10 mM, black; 20 mM, red; 30 mM, blue; 40 mM, green; 50 mM, cyan). Scan Rate: 100 mV/s with a glassy carbon working electrode. (b) Plot of i_c vs. [TFA].

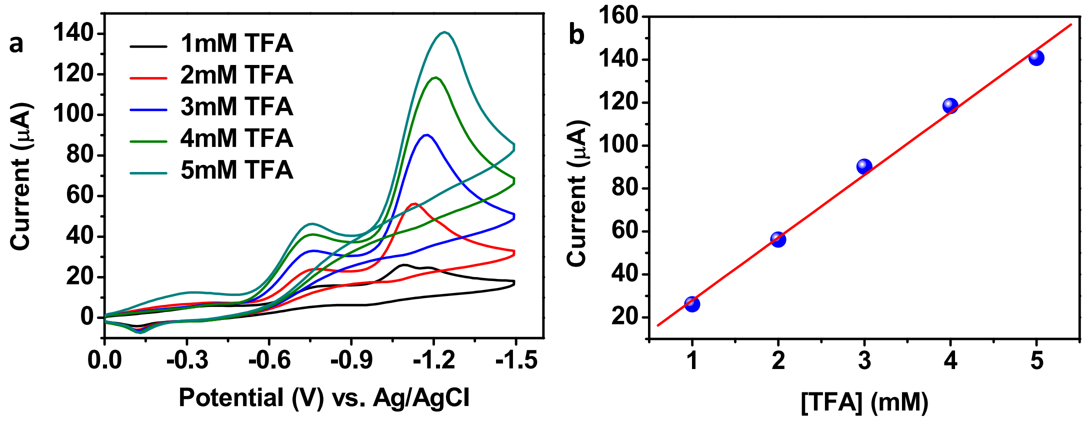

Supplementary Figure 14. (a) Cyclic voltammograms of 0.10 mM Co_3**TPS**$_2$ in DMF solution containing 0.1 M $TBAPF_6$ upon the addition of trifluoroacetic acid (TFA, 1 mM, black; 2 mM, red; 3 mM, blue; 4 mM, green; 5 mM, cyan). Scan Rate: 100 mV/s with a glassy carbon working electrode. (b) Plot of i_c vs. [TFA].

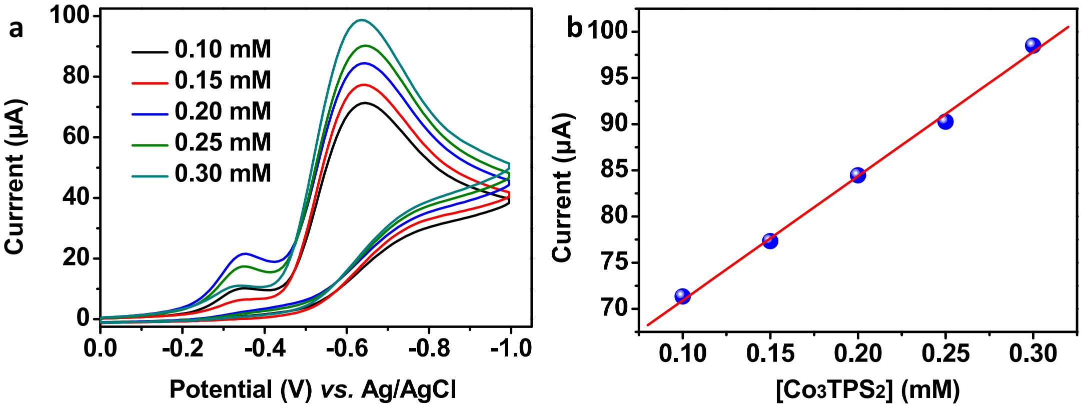

Supplementary Figure 15. (a) Cyclic voltammograms of 6.5 mM *p*-toluenesulfonic acid in DMF solution containing 0.1 M $TBAPF_6$ with different Co_3**TPS**$_2$ concentration (0.10 mM, black; 0.15 mM, red; 0.20 mM, blue; 0.25 mM, green; 0.30 mM, cyan). Scan rate: 100 mV/s with a glassy carbon working electrode. (b) Plot of i_c vs. [Co_3**TPS**$_2$].

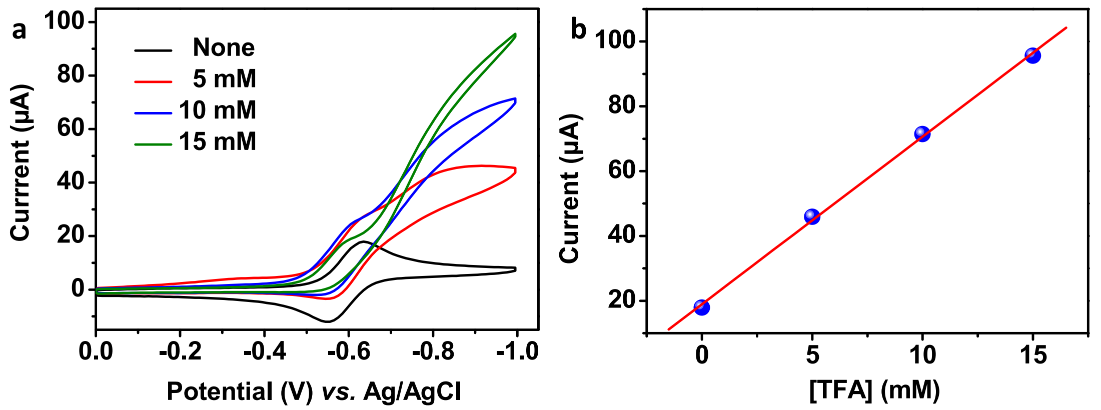

Supplementary Figure 16. (a) Cyclic voltammograms of 0.10 mM Co**BDT**$_2$ in DMF solution containing 0.1 M TBAPF$_6$ upon the addition of trifluoroacetic acid (TFA, none, black; 5 mM, red; 10 mM, blue; 15 mM, green). Scan Rate: 100 mV/s with a glassy carbon working electrode. (b) Plot of i_c vs. [TFA].

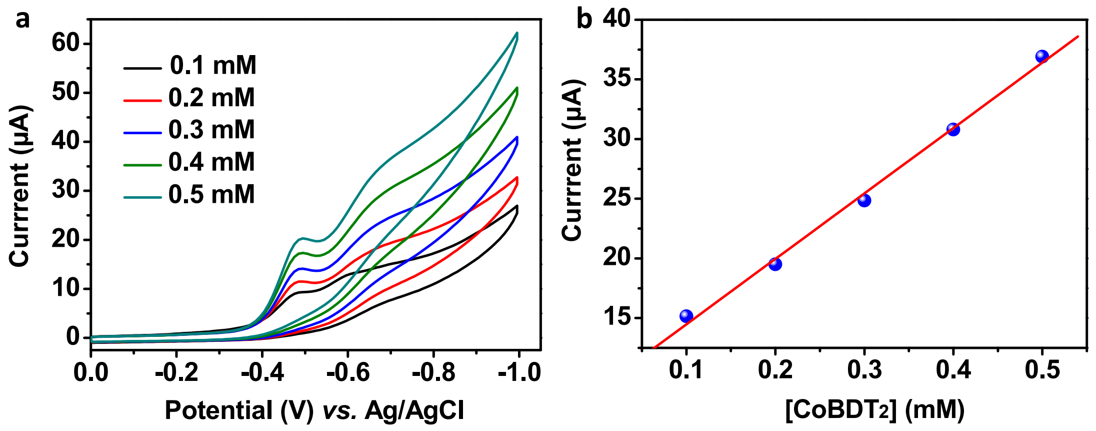

Supplementary Figure 17. (a) Cyclic voltammograms of 6.5 mM *p*-toluenesulfonic acid in DMF solution containing 0.1 M $TBAPF_6$ with different Co**BDT**$_2$ concentration (0.10 mM, black; 0.20 mM, red; 0.30 mM, blue; 0.40 mM, green; 0.50 mM, cyan). Scan rate: 100 mV/s with a glassy carbon working electrode. (b) Plot of i_c vs. [Co**BDT**$_2$].

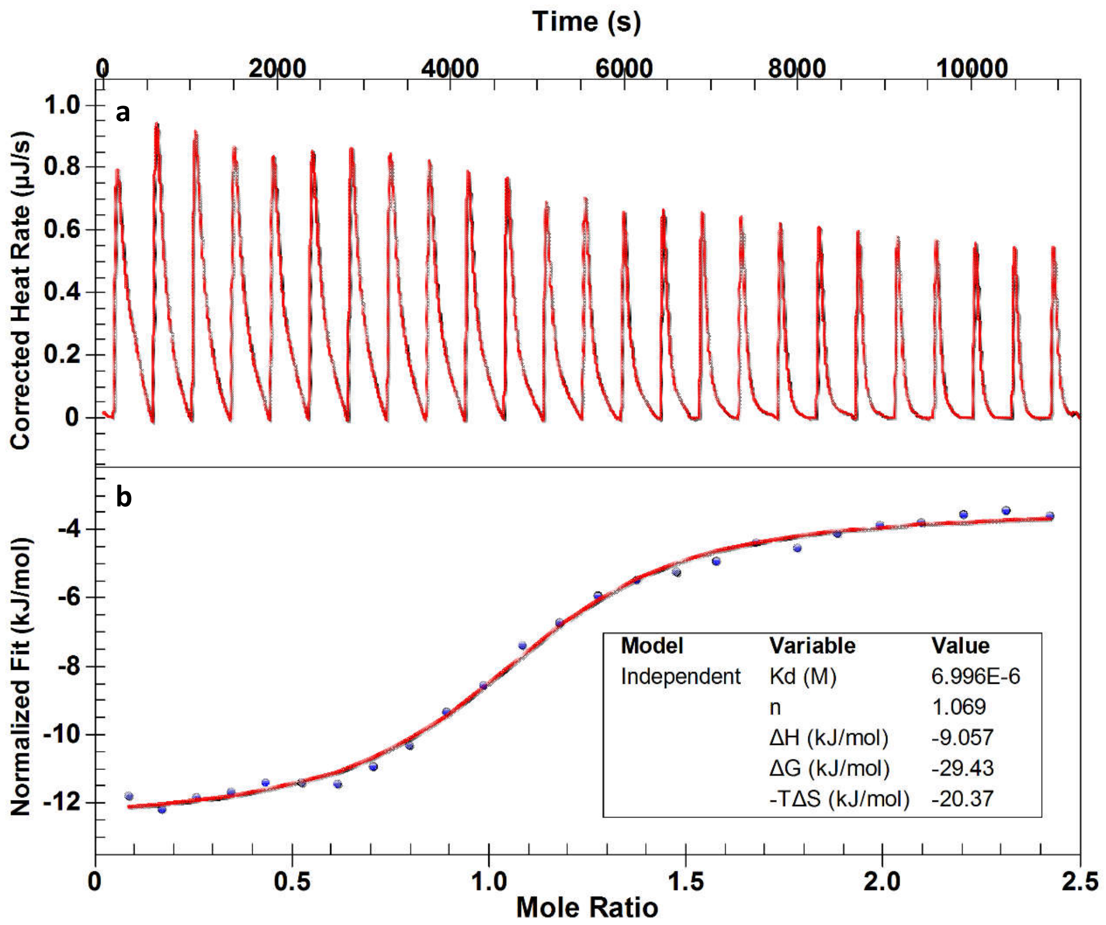

Supplementary Figure 18. The ITC experiments were performed by an isothermal titration microcalorimeter at atmospheric pressure and at 298.15 K, giving the association constants (K) and the thermodynamic parameters. A solution of **PNQ** in a 0.25 mL syringe was sequentially injected with stirring at 250 rpm into a solution of Co_3**TPS**$_2$ in the sample cell (1.30 mL volume). All the thermodynamic parameters reported in this work were obtained by using the 'independent' model.

Microcalorimetric titration of Co_3**TPS**$_2$ with **PNQ** in EtOH/H_2O (v:v = 3:2) solution. (a) Raw data for sequential 25 injections (10 μL per injection) of **PNQ** solution (1.20 mM) injecting into Co_3**TPS**$_2$ solution (0.15 mM). (b) Apparent reaction heat obtained from the integration of calorimetric traces.

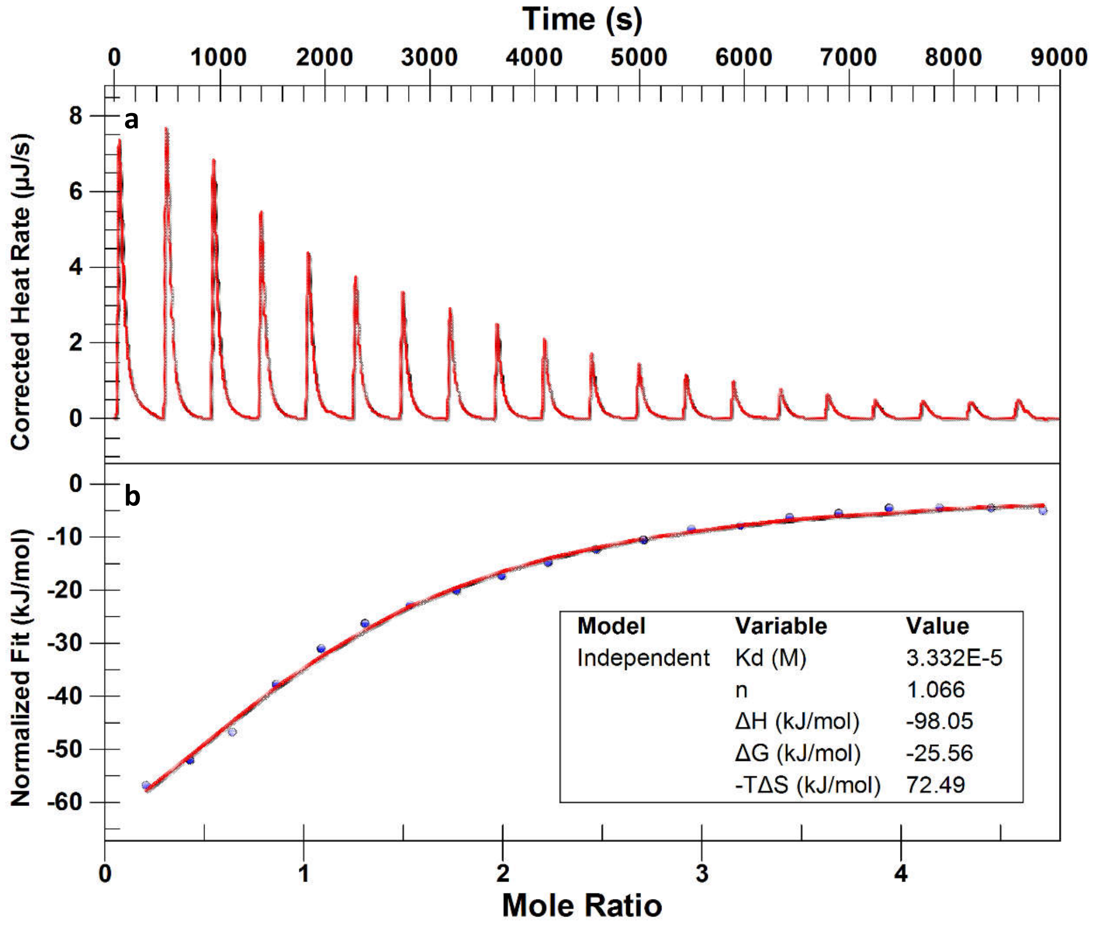

Supplementary Figure 19. The ITC experiments were performed by an isothermal titration microcalorimeter at atmospheric pressure and at 298.15 K, giving the association constants (K) and the thermodynamic parameters. A solution of NADH in a 0.25 mL syringe was sequentially injected with stirring at 250 rpm into a solution of Co_3**TPS**$_2$ ⊃ **PNQ** complexes in the sample cell (1.30 mL volume). All the thermodynamic parameters reported in this work were obtained by using the 'independent' model.

Microcalorimetric titration of Co_3**TPS**$_2$/**PNQ** with NADH in EtOH/H_2O (v:v = 3:2) solution. (a) Raw data for sequential 25 injections (10 µL per injection) of NADH (1.0 mM) injecting into a mixed solution of Co_3**TPS**$_2$ (0.05 mM) and **PNQ** (0.05 mM). (b) Apparent reaction heat obtained from the integration of calorimetric traces.

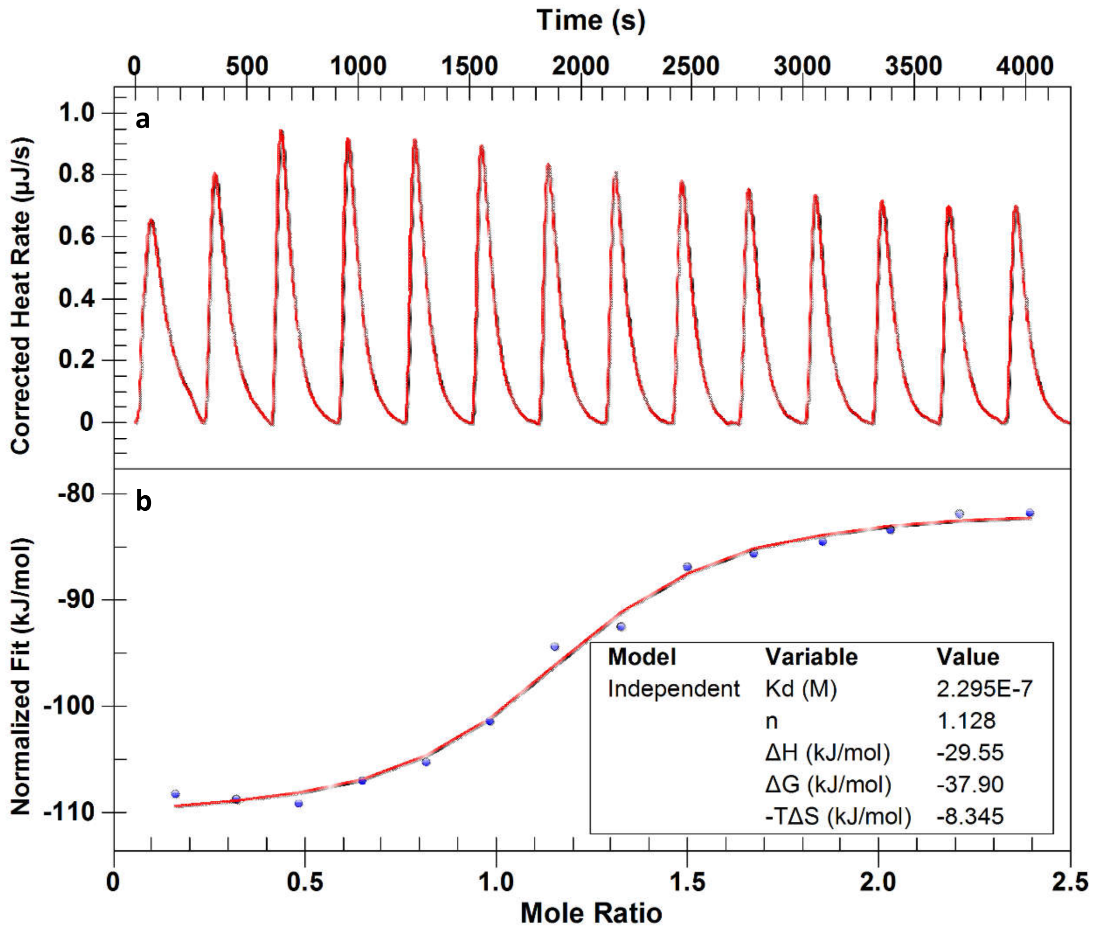

Supplementary Figure 20. The ITC experiments were performed by an isothermal titration microcalorimeter at atmospheric pressure and at 298.15 K, giving the association constants (K) and the thermodynamic parameters. A solution of ADH in a 0.25 mL syringe was sequentially injected with stirring at 250 rpm into a solution of Co_3**TPS**$_2$ in the sample cell (1.30 mL volume). All the thermodynamic parameters reported in this work were obtained by using the ‘independent’ model.

Microcalorimetric titration of Co_3**TPS**$_2$ with ADH in EtOH/H_2O (v:v = 3:2) solution. (a) Raw data for sequential 25 injections (10 μL per injection) of ADH solution (75.0 μM) injecting into Co_3**TPS**$_2$ solution (5.0 μM). (b) Apparent reaction heat obtained from the integration of calorimetric traces.

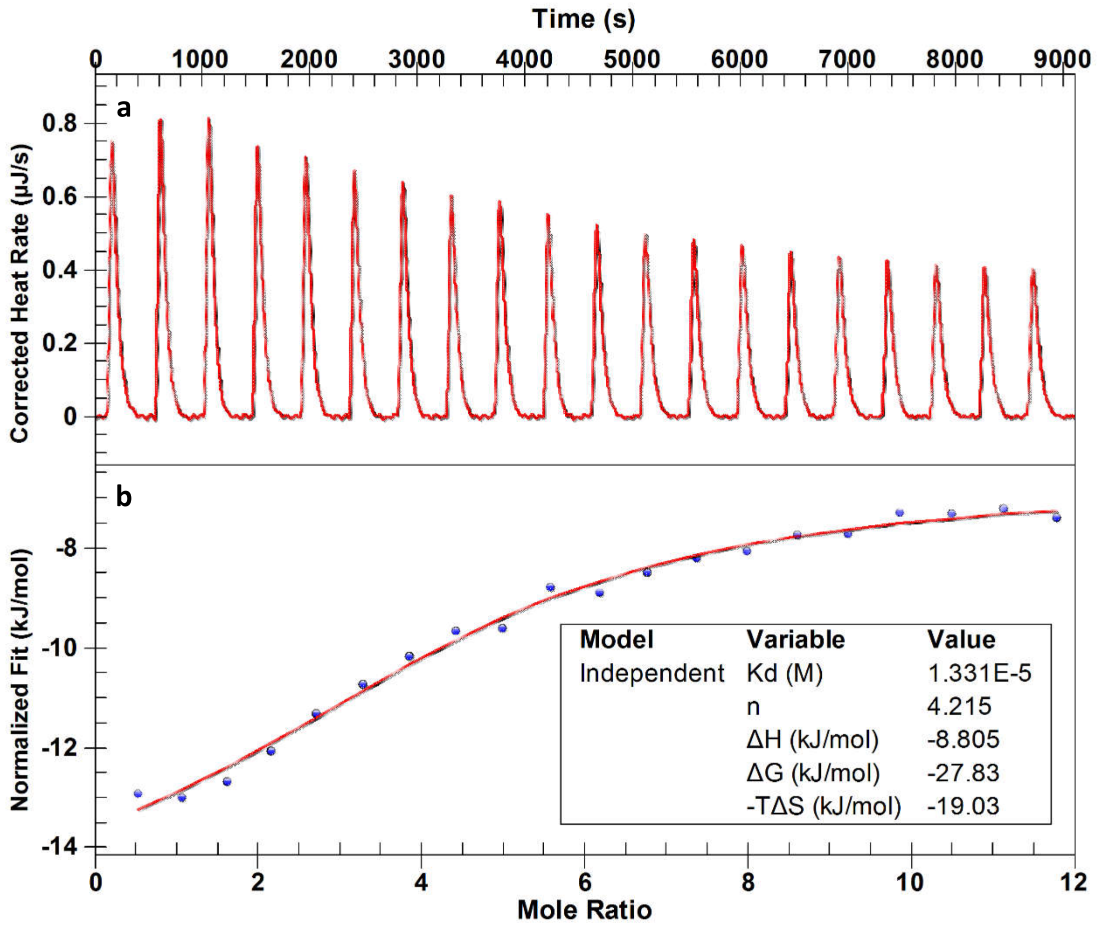

Supplementary Figure 21. The ITC experiments were performed by an isothermal titration microcalorimeter at atmospheric pressure and at 298.15 K, giving the association constants (K) and the thermodynamic parameters. A solution of Co**BDT**$_2$ in a 0.25 mL syringe was sequentially injected with stirring at 250 rpm into a solution of ADH in the sample cell (1.30 mL volume). All the thermodynamic parameters reported in this work were obtained by using the 'independent' model.

Microcalorimetric titration of Co**BDT**$_2$ with ADH in EtOH/H_2O (v:v = 3:2) solution. (a) Raw data for sequential 25 injections (10 μL per injection) of Co**BDT**$_2$ solution (0.5 mM) injecting into ADH solution (10.0 μM). (b) Apparent reaction heat obtained from the integration of calorimetric traces.

Data Relative to Catalysis

Supplementary Table 3. Metal-organic complexes as catalyst for NADH-mediated hydrogen production.[a]

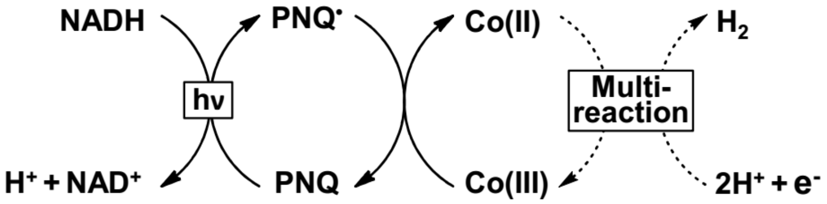

Entry	Catalyst	loading (μM)	**PNQ** (mM)	NADH (mM)	H_2 (μL)
1	Co_3**TPS**$_2$	20.0	0.5	2.0	40
2	Co_3**TPS**$_2$	—	0.5	2.0	0
3	Co_3**TPS**$_2$	20.0	—	2.0	0
4	Co_3**TPS**$_2$	20.0	0.5	—	0
5[b]	Co_3**TPS**$_2$	20.0	0.5	2.0	0
6[c]	Co_3**TPS**$_2$	20.0	0.5	2.0	11
7	Co**BDT**$_2$	20.0	0.5	2.0	19
8	Co**BDT**$_2$	60.0	0.5	2.0	32

[a]Reaction conditions: EtOH/H_2O (v:v = 3:2, pH 4.5), Xe 300W, 4 h. The amount of hydrogen was determined by GC with an external standard method. [b]In the absence of light. [c]In the presence of inhibitor **DTQ** (0.1 M).

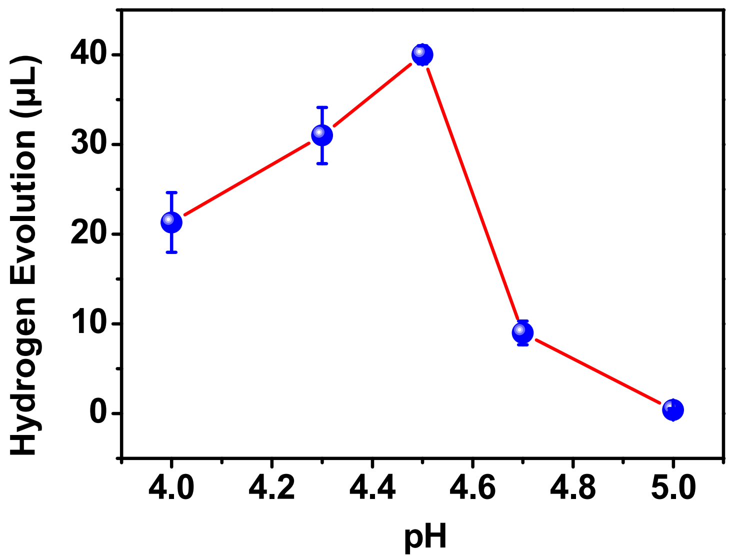

Supplementary Figure 22. Photocatalytic hydrogen evolution in EtOH/H_2O (v:v = 3:2) of the systems containing Co_3**TPS**$_2$ (20.0 μM), **PNQ** (0.5 mM) and NADH (2.0 mM) at different pH value. Data points and error bars represent the mean ± s.d. of three independent experiments.

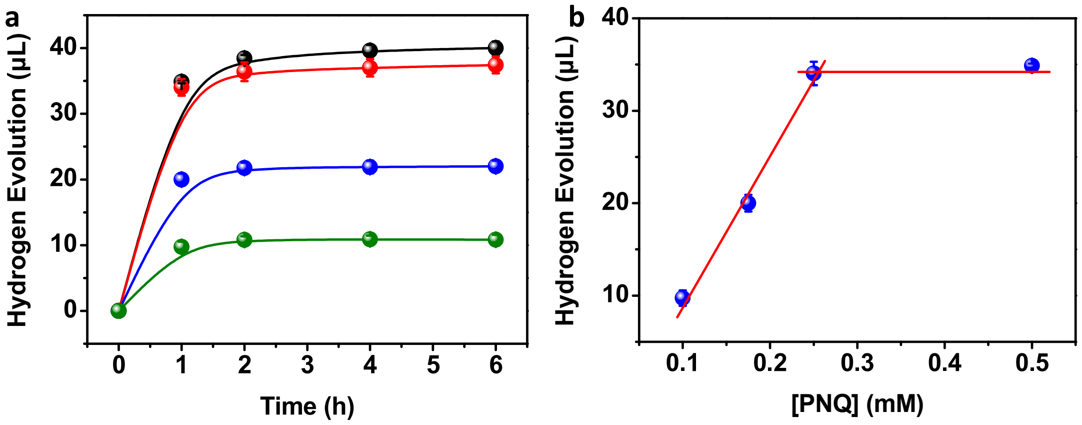

Supplementary Figure 23. (a) Photocatalytic hydrogen evolution in EtOH/H_2O (v:v = 3:2) of the systems containing Co_3**TPS**$_2$ (20.0 μM) and NADH (2.0 mM) at pH 4.5 with various **PNQ** concentration. (b) The picture shows the initial rate *vs.* content of **PNQ**. Data points and error bars represent the mean ± s.d. of three independent experiments in both figures.

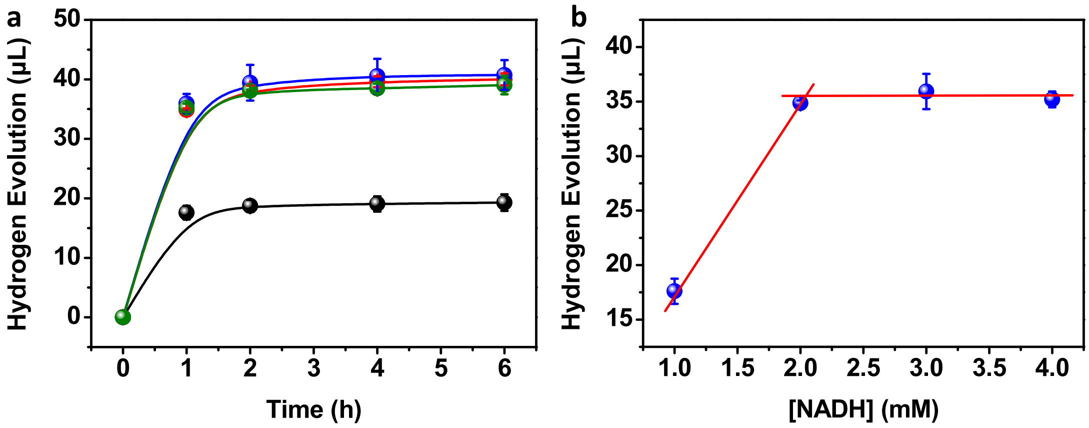

Supplementary Figure 24. (a) Photocatalytic hydrogen evolution in EtOH/H_2O (v:v = 3:2) of the systems containing Co_3**TPS**$_2$ (20.0 μM) and **PNQ** (0.5 mM) at pH 4.5 with various NADH concentration. (b) The picture shows the initial rate *vs.* content of NADH. Data points and error bars represent the mean ± s.d. of three independent experiments in both figures.

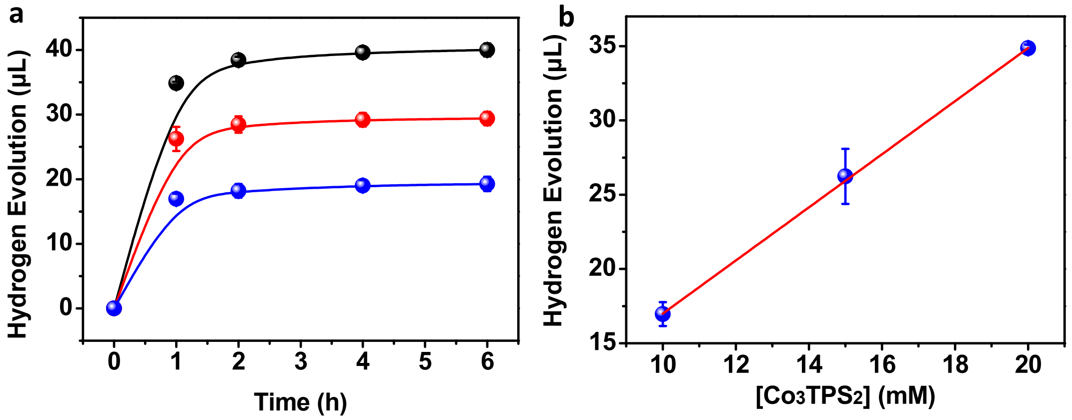

Supplementary Figure 25. (a) Photocatalytic hydrogen evolution in EtOH/H_2O (v:v = 3:2) of the systems containing **PNQ** (0.5 mM) and NADH (2.0 mM) at pH 4.5 with various Co_3**TPS**$_2$ concentration. (b) The picture shows the initial rate *vs.* content of Co_3**TPS**$_2$. Data points and error bars represent the mean ± s.d. of three independent experiments in both figures.

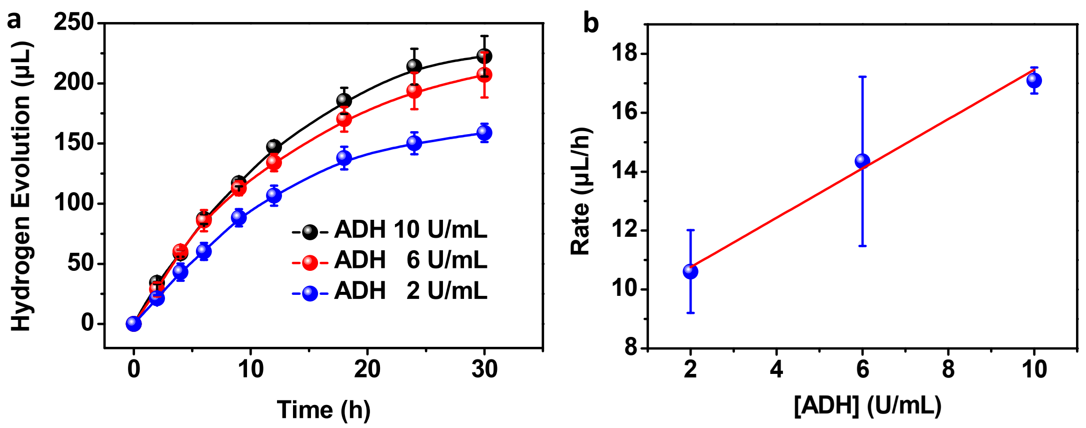

Supplementary Figure 26. (a) Photocatalytic hydrogen evolution in EtOH/H_2O (v:v = 3:2, pH 4.5) of the systems containing 2.0 mM NAD^+, 20.0 μM Co_3**TPS**$_2$ and 0.5 mM **PNQ** with various ADH content. (b) The picture shows the initial rate *vs.* content of ADH. Data points and error bars represent the mean ± s.d. of three independent experiments in both figures.

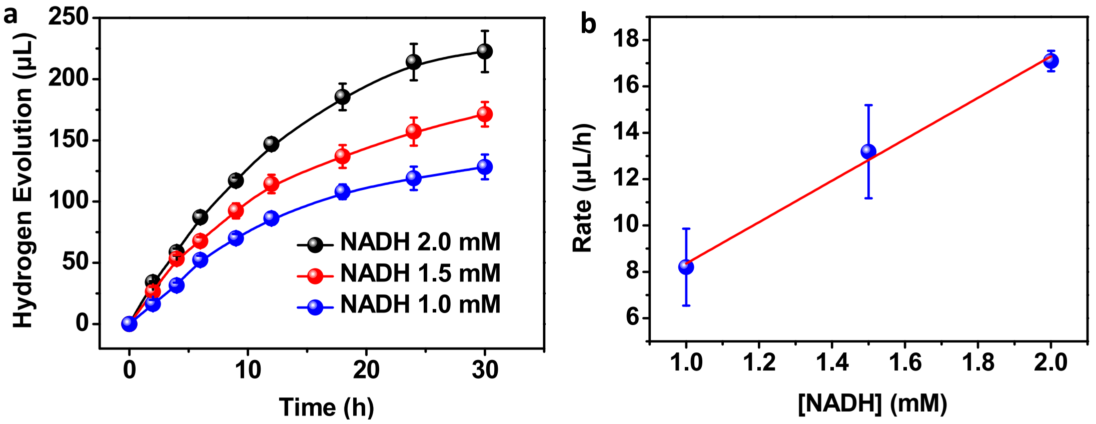

Supplementary Figure 27. (a) Photocatalytic hydrogen evolution in EtOH/H_2O (v:v = 3:2, pH 4.5) of the systems containing 10 U·mL^{-1} ADH, 20.0 μM Co_3**TPS**$_2$ and 0.5 mM **PNQ** with various NAD^+ concentration. (b) The picture shows the initial rate *vs.* concentration of NAD^+. Data points and error bars represent the mean ± s.d. of three independent experiments in both figures.

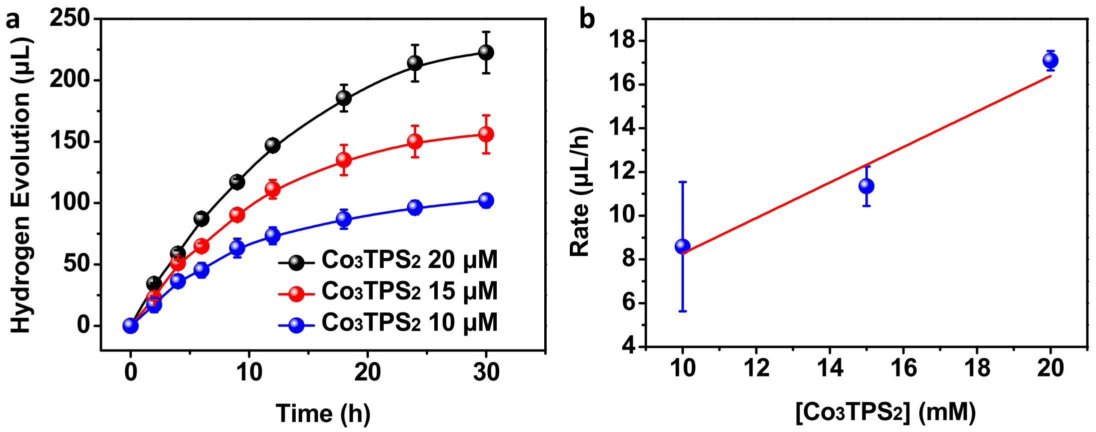

Supplementary Figure 28. (a) Photocatalytic hydrogen evolution in EtOH/H_2O (v:v = 3:2, pH 4.5) of the systems containing 10 U·mL^{-1} ADH, 2.0 mM NAD^+ and 0.5 mM **PNQ** with various Co_3**TPS**$_2$ concentration. (b) The picture shows the initial rate *vs.* concentration of Co_3**TPS**$_2$. Data points and error bars represent the mean ± s.d. of three independent experiments in both figures.

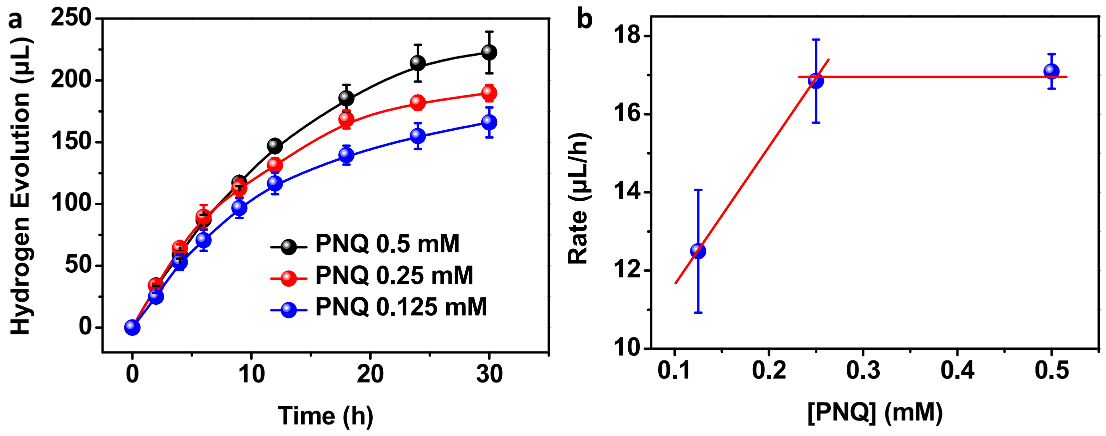

Supplementary Figure 29. (a) Photocatalytic hydrogen evolution in EtOH/H_2O (v:v = 3:2, pH 4.5) of the systems containing 10 U·mL^{-1} ADH, 2.0 mM NAD^+ and 20.0 μM Co_3**TPS_2** with various **PNQ** concentration. (b) The picture shows the initial rate *vs.* concentration of **PNQ**. Data points and error bars represent the mean ± s.d. of three independent experiments in both figures.

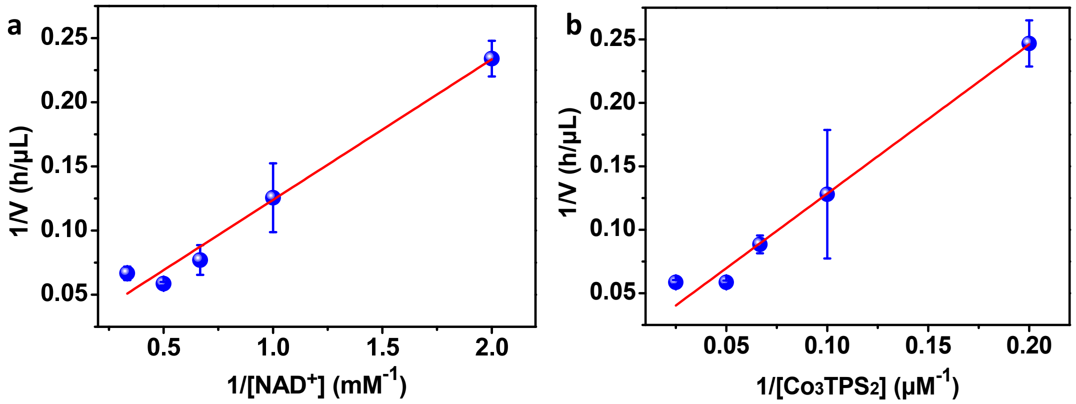

Supplementary Figure 30. (a) Double reciprocal plot of NAD^+ dependent photocatalytic kinetics of the system containing 10 U·mL^{-1} ADH, 20.0 μM Co_3**TPS_2** and 0.5 mM **PNQ** in EtOH/H_2O (v:v = 3:2, pH 4.5). (b) Double reciprocal plot of Co_3**TPS_2** dependent photocatalytic kinetics of the system containing 10 U·mL^{-1} ADH, 2.0 mM NAD^+ and 0.5 mM **PNQ** in EtOH/H_2O (v:v = 3:2, pH 4.5). Data points and error bars represent the mean ± s.d. of three independent experiments in both figures.

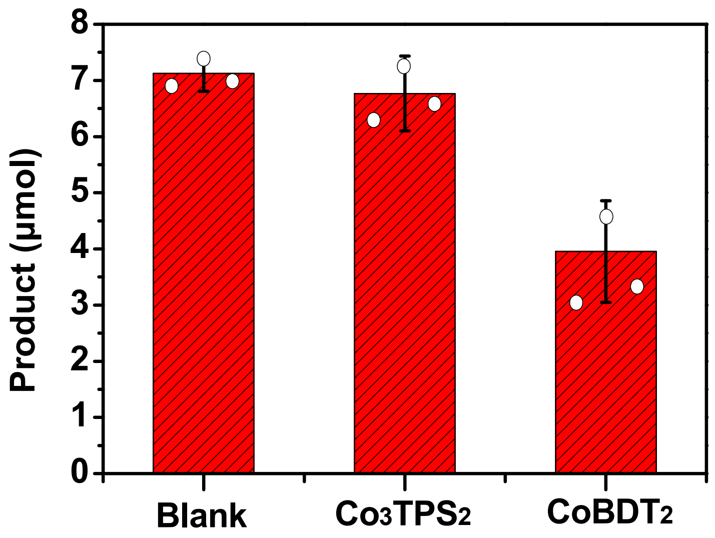

Supplementary Figure 31. Aldehyde production in the systems containing 2.0 mM NAD^+, 10 $U{\cdot}mL^{-1}$ ADH in the presence/absence of catalyst (20.0 μM for Co_3**TPS**$_2$ or 60.0 μM for Co**BDT**$_2$) in EtOH/H_2O (v:v = 3:2, pH 4.5) within 12 h. Data points and error bars represent the mean ± s.d. of three independent experiments.

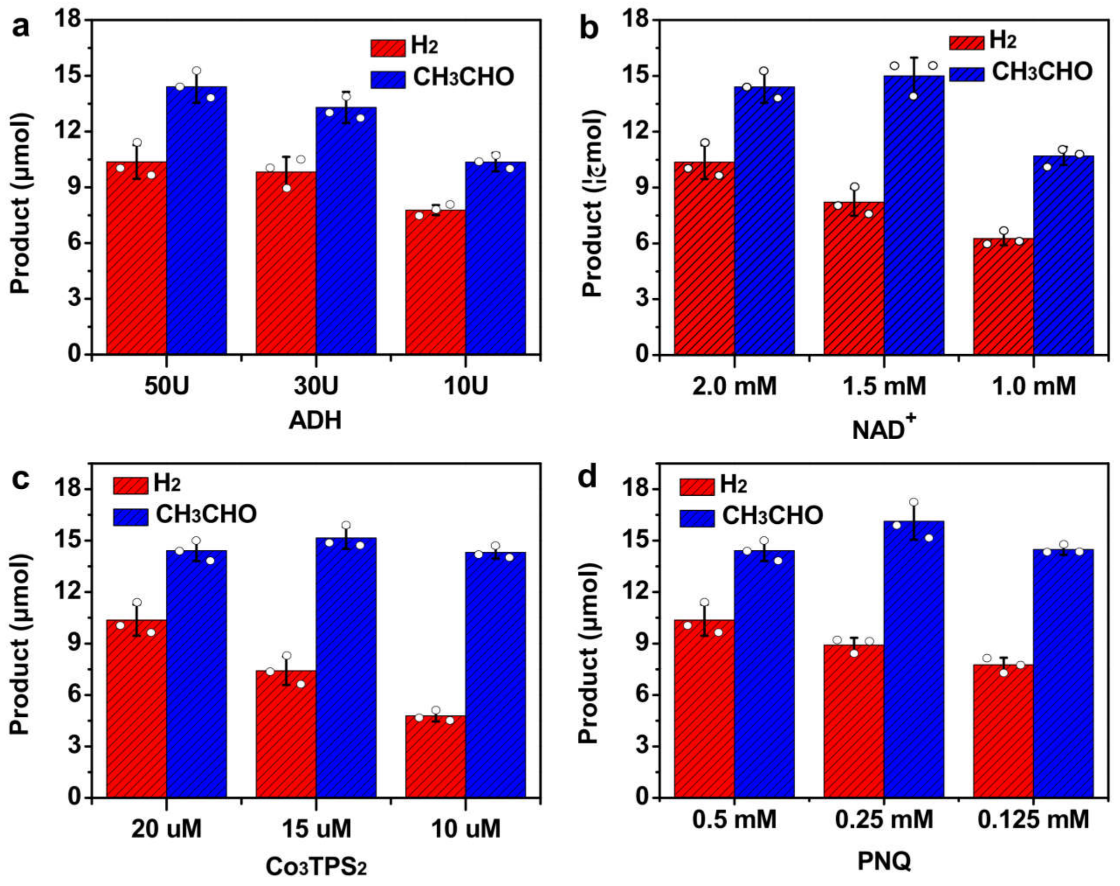

Supplementary Figure 32. Photocatalytic alcohol splitting in the systems containing (a) 2.0 mM NAD^+, 20.0 μM Co_3**TPS**$_2$ and 0.5 mM **PNQ** with various ADH content; (b) 10 U·mL^{-1} ADH, 20.0 μM Co_3**TPS**$_2$ and 0.5 mM **PNQ** with various NAD^+ concentration; (c) 10 U·mL^{-1} ADH, 2.0 mM NAD^+ and 0.5 mM **PNQ** with various Co_3**TPS**$_2$ concentration; (d) 10 U·mL^{-1} ADH, 2.0 mM NAD^+ and 20.0 μM Co_3**TPS**$_2$ with various **PNQ** concentration. All experiments performed in EtOH/H_2O (v:v = 3:2, pH 4.5) within 42 h. Data points and error bars represent the mean ± s.d. of three independent experiments in all figures.

General information for theoretical 'docking study'

Docking calculations were performed with the AutoDock program 4.2. The Co_3**TPS**$_2$, **PNQ** and NADH were downloaded from the CCDC database. The structure of enzyme alcohol dehydrogenase (PDB code: 5ENV) was downloaded from the PDB database. The cage Co_3**TPS**$_2$ was used to perform the docking calculation after energy minimization. The models of the enzyme were refined by removing hydrogen atoms. Polar hydrogens were then added, followed by assignment of Kollman charges, fragmental volumes, and atomic solvation parameters to adhesive by means of AutoDock Tools. For the ligand, the molecule was refined by removing and subsequently adding hydrogen atoms in a similar manner to that for adhesive. Next, Gasteiger partial charges were assigned to the ligands, and nonpolar hydrogens were merged. All torsions were allowed to rotate during docking. The Lamarckian genetic algorithm was used to determine the appropriate binding positions, orientations, and conformations of the ligands. Default parameters were used, except for the number of generations which was set to 300. The blind docking strategy was used with a 50 Å × 78 Å × 114 Å grid box which ensured sufficient spaced to cover the entire surface of the enzyme. The Lamarckian genetic algorithm was chosen with default parameters except for the number of generations, which was set to 100 for more accurate docking results. The best docking mode of the host-guest complex was chosen based on the binding energy score, clustering, and chemical reasonableness.

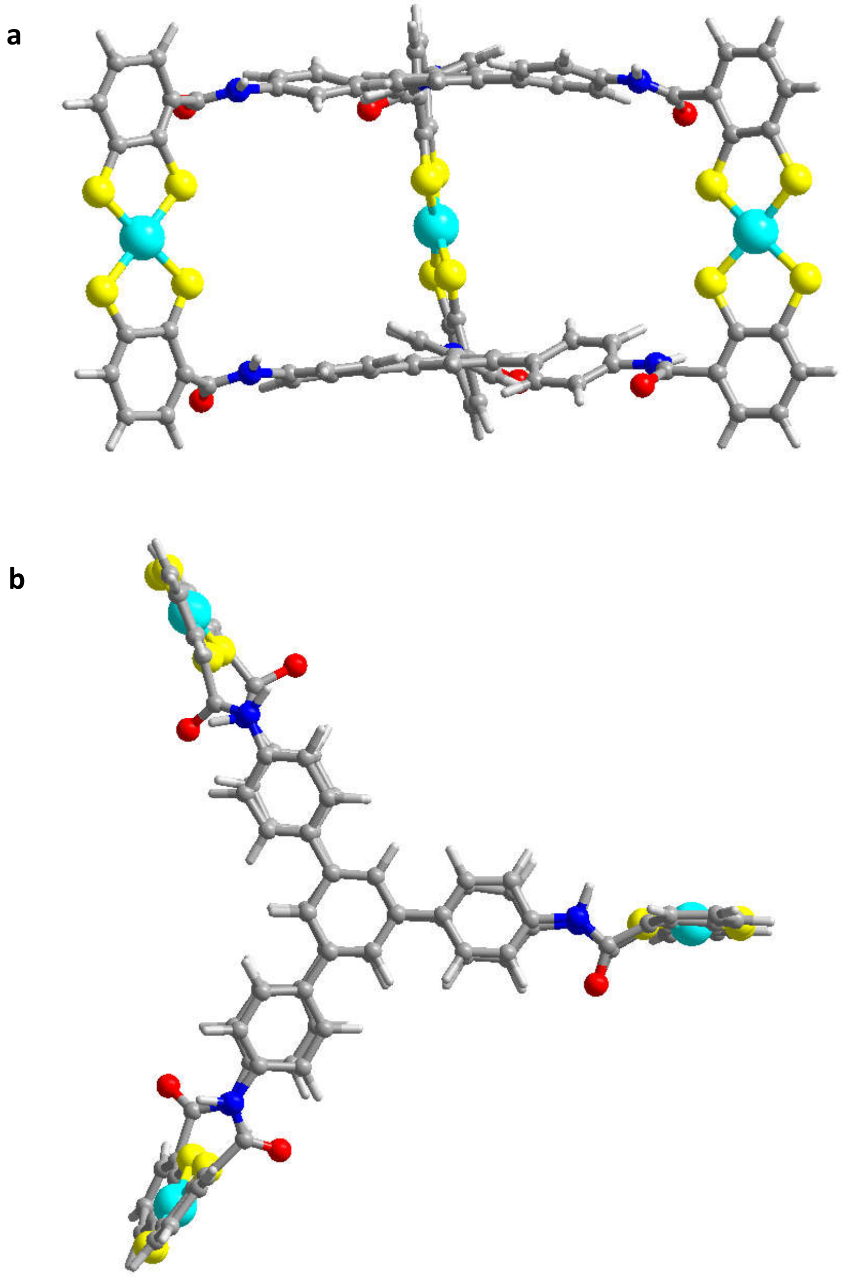

Supplementary Figure 33. (a, b) The theoretical structure of Co_3**TPS**$_2$ after energy minimization consistent with the crystal structure of Co_3**TPS**$_2$.

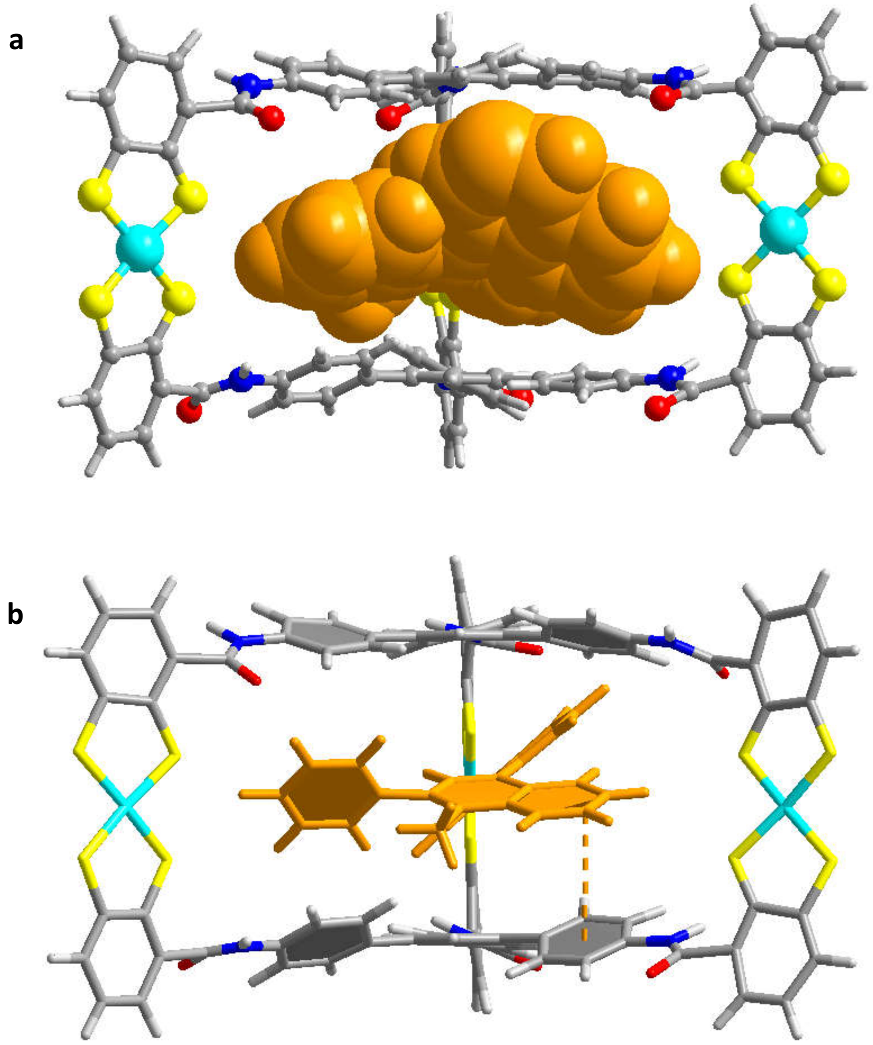

Supplementary Figure 34. (a, b) Theoretical 'docking study' optimized model of Co_3**TPS**$_2$ $\supset$ **PNQ** complex, showing that **PNQ** existed in the Co_3**TPS**$_2$ cavity with the assist of aromatic stacking interactions.

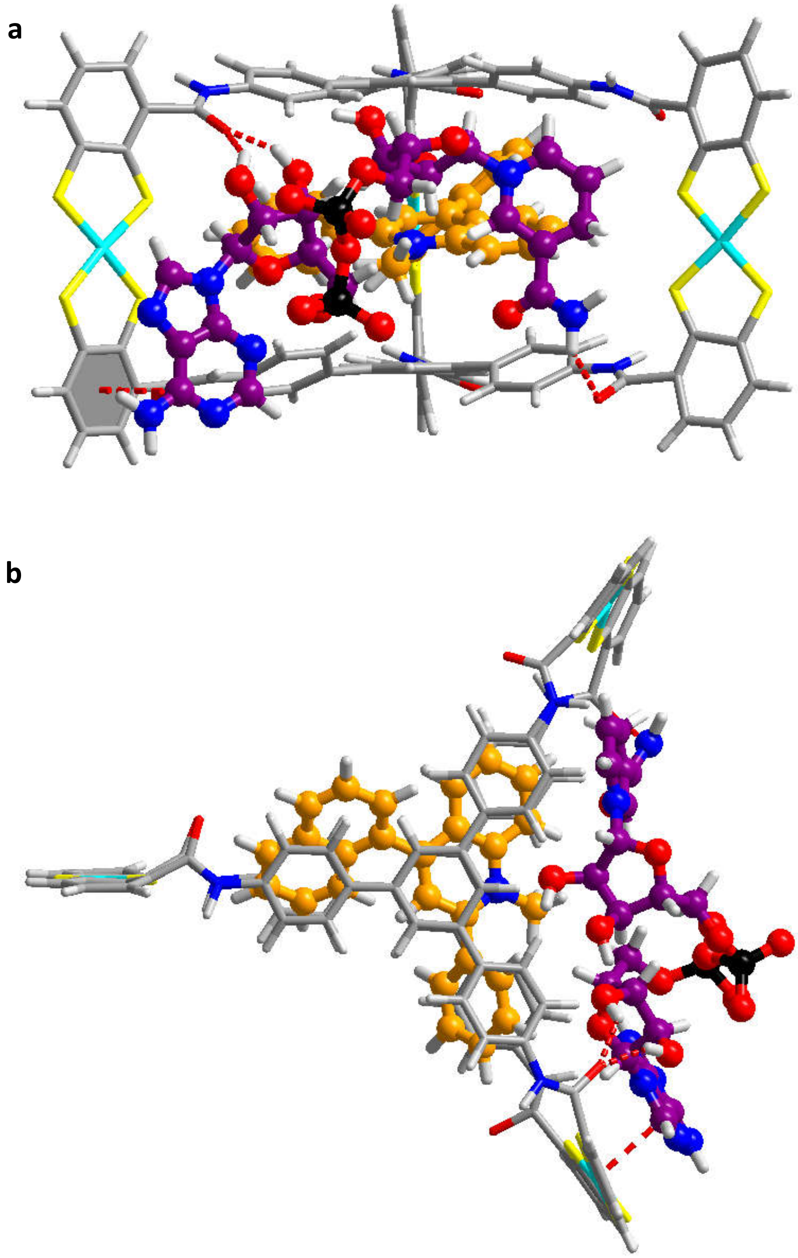

Supplementary Figure 35. (a, b) Theoretical 'docking study' optimized model of Co_3**TPS**$_2$ ⊃ **PNQ** and NADH, showing NADH was located at the opening window of Co_3**TPS**$_2$ due to the multiple hydrogen bonds on the artificial host and formed an integrated superstructure.

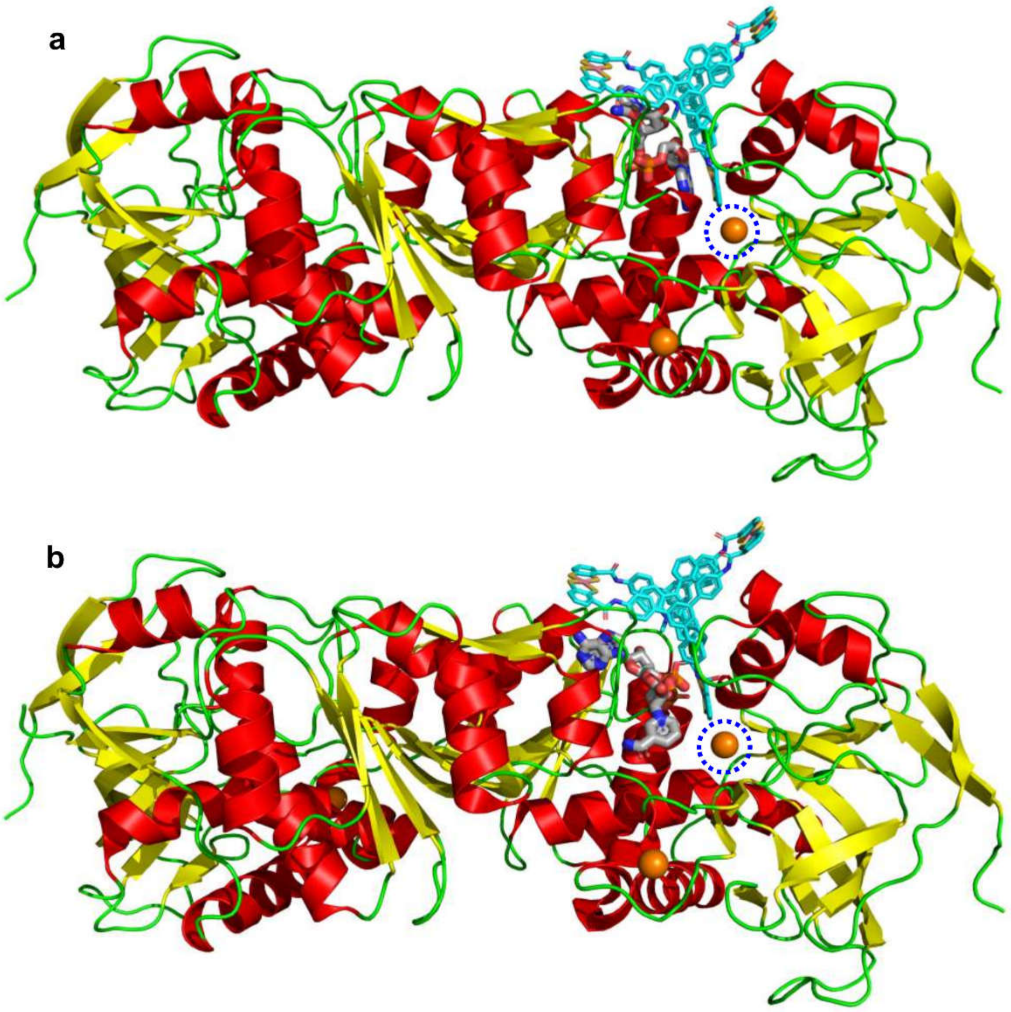

Supplementary Figure 36. (a) Theoretical 'docking study' optimized model of Co_3**TPS**$_2$ ⊃ **PNQ** and NADH with enzyme ADH. (b) Theoretical 'docking study' optimized model of Co_3**TPS**$_2$ ⊃ **PNQ** complex with enzyme ADH containing NAD^+. The active centers of ADH were marked by blue circle. These results showed that the dye-containing cage bound to the catalytic pocket of the enzyme ADH, and the coenzyme NADH was locked at the ADH pocket approaching to the artificial and natural catalytic sites.

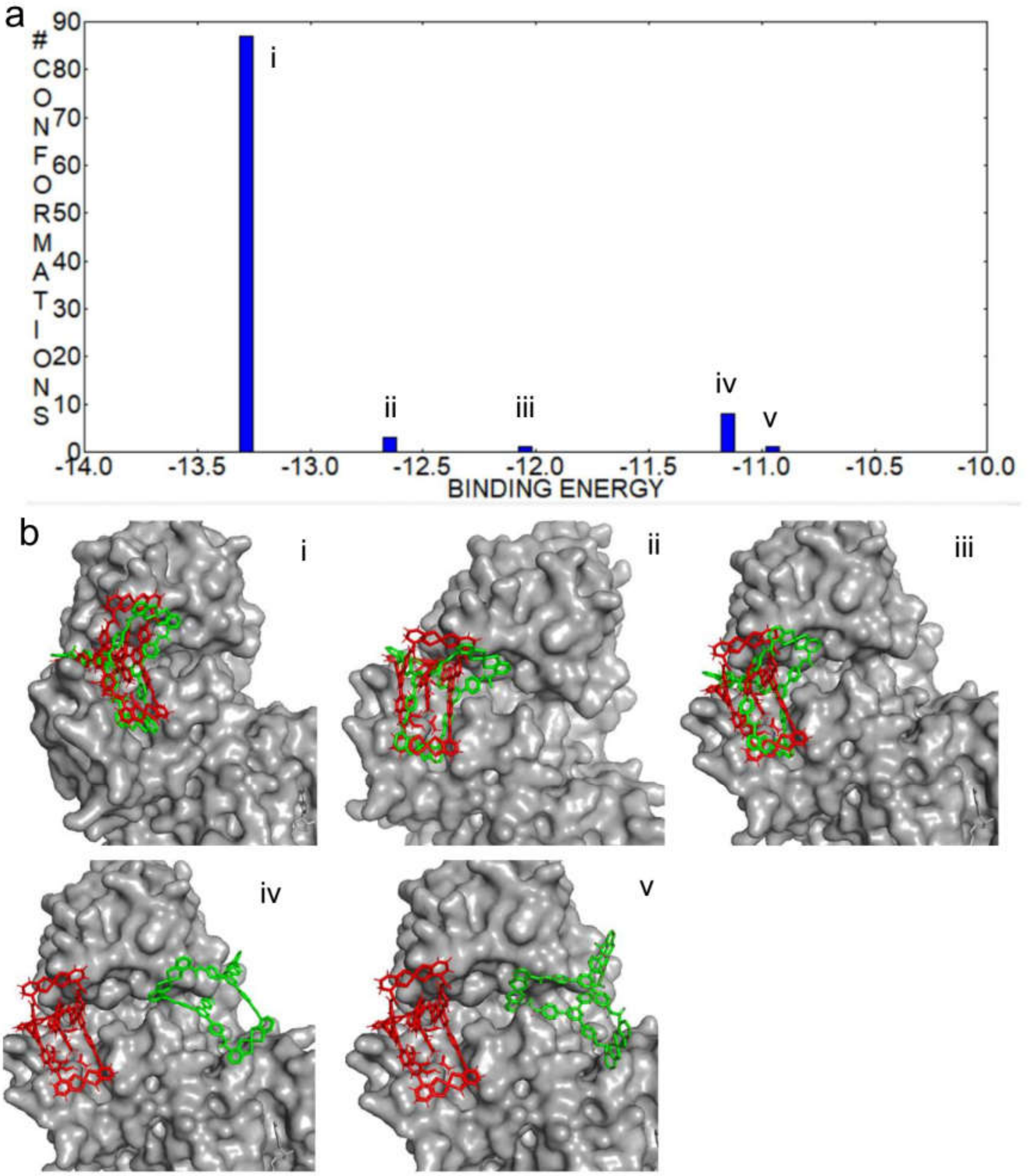

Supplementary Figure 37. (a) Cluster map related to theoretical 'docking study' model of Co_3**TPS**$_2$ with enzyme ADH in a random binding mode, showing the binding energy in different binding modes. (b) Theoretical 'docking study' model showed five binding modes of Co_3**TPS**$_2$ with enzyme ADH (i to v), the green part represented the actual location of the cage and the red part showed the location of the dye-containing cage in the optimized model.

Supplementary References

1. Dong, J., Wang, M., Zhang, P., Yang, S., Liu, J., Li, X. & Sun, L. C. Promoting effect of electrostatic interaction between a cobalt catalyst and a xanthene dye on visible-light-driven electron transfer and hydrogen production. *J. Phys. Chem. C* **115**, 15089–15096 (2011).

2. Simon, T., Bouchonville, N., Berr, M. J., Vaneski, A., Adrović, A., Volbers, D., Wyrwich, R., Döblinger, M., Susha, A. S., Rogach, A. L., Jäckel, F., Stolarczyk, J. K. & Feldmann, J. Redox shuttle mechanism enhances photocatalytic H_2 generation on Ni-decorated CdS nanorods. *Nat. Mater.* **13**, 1013−1018 (2014).

3. Liu, K., Yuan, C., Zou, Q., Xie, Z. & Yan, X. A Self-assembled zinc/cystine-based chloroplast mimics capable of photoenzymatic reactions for sustainable fuel synthesis. *Angew. Chem. Int. Ed.* **56**, 7876−7880 (2017).

4. Prier, C. K., Rankic, D. A. & MacMillan, D. W. C. Visible light photoredox catalysis with transition metal complexes: applications in organic synthesis. *Chem. Rev.* **113**, 5322−5363 (2013).

5. Thordarson, P. Determining association constants from titration experiments in supramolecular chemistry. *Chem. Soc. Rev.* **40**, 1305−1323 (2011).

6. Connors, K. A. *Binding constants* (John Wiley, New York, 1987).

7. Yamada, Y., Miyahigashi, T., Kotani, H., Ohkubo, K. & Fukuzumi, S. Photocatalytic hydrogen evolution under highly basic conditions by using Ru nanoparticles and 2-phenyl-4-(1-naphthyl) quinolinium ion. *J. Am. Chem. Soc.* **133**, 16136−16145 (2011).

8. McNamara, W. R., Han, Z., Alperin, P. J., Brennessel, W. W., Holland, P. L. & Eisenberg, R. A cobalt–dithiolene complex for the photocatalytic and electrocatalytic reduction of protons. *J. Am. Chem. Soc.* **133**, 15368−15371 (2011).

9. Birkmann, B., Fröhlich, R. & Hahn, F. E. Assembly of a tetranuclear host with a tris(benzene-*o*-dithiolato) ligand. *Chem. Eur. J.* **15**, 9325−9329 (2009).

10. Okamura, T., Kunisue, K., Omi, Y. & Onitsuka, K. Strong NH···S hydrogen bonds in molybdoenzyme models containing anilide moieties. *Dalton Trans.* **42**, 7569–7578 (2013).

11. SMART, Data collection software (version 5.629) (Bruker AXS Inc., Madison, WI, 2003).

12. SAINT, Data reduction software (version 6.45) (Bruker AXS Inc.; Madison, WI, 2003).

13. Sheldrick, G. M. SHELXTL97, Program for Crystal Structure Solution (University of Göttingen: Göttingen, Germany, 1997).

14. Spek, A. L. Single-crystal structure validation with the program PLATON. *J. Appl. Cryst.* **36***,* 7−13 (2003).
